# Conflicting Selection Pressures Will Constrain Viral Escape from Interfering Particles: Principles for Designing Resistance-Proof Antivirals

**DOI:** 10.1371/journal.pcbi.1004799

**Published:** 2016-05-06

**Authors:** Luke I. Rast, Igor M. Rouzine, Ganna Rozhnova, Lisa Bishop, Ariel D. Weinberger, Leor S. Weinberger

**Affiliations:** 1 Gladstone Institutes (Virology and Immunology), San Francisco, California, United States of America; 2 Wyss Institute for Biologically Inspired Engineering, Harvard University, Boston, Massachusetts, United States of America; 3 Department of Biochemistry and Biophysics, University of California, San Francisco, San Francisco, California, United States of America; 4 QB3: California Institute for Quantitative Biosciences, University of California, San Francisco, San Francisco, California, United States of America; ETH Zurich, SWITZERLAND

## Abstract

The rapid evolution of RNA-encoded viruses such as HIV presents a major barrier to infectious disease control using conventional pharmaceuticals and vaccines. Previously, it was proposed that defective interfering particles could be developed to indefinitely control the HIV/AIDS pandemic; in individual patients, these engineered molecular parasites were further predicted to be refractory to HIV’s mutational escape (i.e., be ‘resistance-proof’). However, an outstanding question has been whether these engineered interfering particles—termed Therapeutic Interfering Particles (TIPs)—would remain resistance-proof at the population-scale, where TIP-resistant HIV mutants may transmit more efficiently by reaching higher viral loads in the TIP-treated subpopulation. Here, we develop a multi-scale model to test whether TIPs will maintain indefinite control of HIV at the population-scale, as HIV (‘unilaterally’) evolves toward TIP resistance by limiting the production of viral proteins available for TIPs to parasitize. Model results capture the existence of two intrinsic evolutionary tradeoffs that collectively prevent the spread of TIP-resistant HIV mutants in a population. First, despite their increased transmission rates in TIP-treated sub-populations, unilateral TIP-resistant mutants are shown to have reduced transmission rates in TIP-untreated sub-populations. Second, these TIP-resistant mutants are shown to have reduced growth rates (i.e., replicative fitness) in both TIP-treated and TIP-untreated individuals. As a result of these tradeoffs, the model finds that TIP-susceptible HIV strains continually outcompete TIP-resistant HIV mutants at both patient and population scales when TIPs are engineered to express >3-fold more genomic RNA than HIV expresses. Thus, the results provide design constraints for engineering population-scale therapies that may be refractory to the acquisition of antiviral resistance.

## Author Summary

A major obstacle to effective antimicrobial therapy campaigns is the rapid evolution of drug resistance. Given the *static* nature of current pharmaceuticals and vaccines, natural selection inevitably drives pathogens to mutate into drug-resistant variants that can resume productive replication. Further, these drug-resistant mutants transmit across populations, resulting in untreatable epidemics. Recently, a therapeutic strategy was proposed in which viral deletion mutants—termed therapeutic interfering particles (TIPs)—are engineered to only replicate by stealing their missing proteins from full-length viruses in co-infected cells. By stealing essential viral proteins, these engineered molecular parasites have been predicted to reduce viral levels in patients and viral transmission events across populations. Yet, a critical question is whether rapidly mutating viruses like HIV can evolve around TIP control by reducing production of the proteins that TIPs must steal in order to replicate (i.e., by ‘starving’ the TIPs). Here we develop a multi-scale model that tests whether TIP-starving HIV mutants can spread across populations to undermine TIP therapy campaigns at the population-scale. Strikingly, model results show that inherent evolutionary tradeoffs prevent these TIP-resistant HIV mutants from increasing in frequency (i.e., these TIP-resistant HIV mutants are continually outcompeted by TIP-sensitive mutants in both patients and populations). Maintained by natural selection, TIPs may offer a novel therapeutic approach to indefinitely control rapidly evolving viral pandemics.

## Introduction

Defective interfering particles (DIPs) are ‘cheaters’ in a viral population. Rather than carrying a full set of genes essential for their own replication, these deletion mutants require co-infection by replication-competent ‘helper’ viruses to provide their missing components for replication, packaging, and spread [1, 2]. By stealing essential viral components from wild-type viruses, DIPs act as molecular parasites of viruses. Further, natural DIPs have been observed to arise spontaneously across a range of viruses, and have been predicted to reduce disease virulence by interfering with viral replication processes [3–9]. Consequently, DIPs have been proposed as novel antiviral therapeutics [5, 10–12].

While natural DIPs have never been documented in HIV infections, HIV-derived DIPs have been engineered artificially [10, 13–15] and shown to reduce HIV replication [10, 16, 17]. Here we quantitatively probe a subset of DIPs that are engineered to have basic reproductive ratios greater than 1 during co-infection with HIV (i.e., maintain stable TIP loads) while suppressing HIV viral loads. These stable and suppressive DIPs are termed therapeutic interfering particles (TIPs). Previous mathematical models predicted that TIPs would substantially outperform current state-of-the-art antiretroviral therapy campaigns [12, 18–21]. However, the models did not test whether TIP efficacy would be undermined at the population-scale by the evolution and spread of TIP-resistant HIV mutants.

Since TIP intervention is designed to reduce wild-type HIV viral loads within individual patients, it may pressure HIV to evolve. Specifically, reductions in HIV load correlate with reduced transmission of HIV [22, 23], so any TIP-mediated reductions would create a selection pressure for ‘resistant’ HIV mutants that are not suppressed. Arguably, the most direct way for HIV to evolve TIP-resistance is by reducing the amount of intracellular resource (e.g., capsid proteins) available for TIP parasitism. In that way, HIV may be able to ‘starve’ the parasitic TIP particles of the resources they parasitize. TIP-resistant HIV mutants would then have the potential to outcompete the wild-type HIV strains within a patient and spread through a host population, progressively nullifying the TIP intervention. This is a form of ‘unilateral escape’, evolutionary escape by mutations affecting a feature encoded by the viral genome but not the (reduced) TIP genome.

Recently, we analyzed the unilateral escape dynamics of HIV resistance at the level of an individual patient and found that HIV mutants that starve TIPs would be selected *against* within individual patients [19]. However, given their reduced suppression, these TIP-resistant HIV mutants would likely transmit from infected individuals more efficiently than the wild-type HIV [22]. Thus, even if disfavored in individual patients, TIP-resistant HIV mutants could supplant the wild-type HIV strain at the population-scale, undermining the effectiveness of a TIP therapy campaign.

We sought to test whether TIP-resistant HIV strains would spread through a population. Notably, the transmission of HIV strains through a population is primarily driven by small ‘core groups’ of infected individuals (~1–2% of the population) who engage in high-risk behaviors [24–26]. TIPs similarly concentrate within these high-risk groups, because TIPs spread via the same transmission routes and risk factors as HIV [18]. Further, the increased prevalence of TIPs within high-risk groups increases the selective pressure in favor of TIP resistance in these sub-populations. And even if TIP-resistance initially emerges in a disparate sub-population, transit through high-risk sub-populations is critical for the population-scale spread of TIP-resistant HIV strains. Consequently, we developed a mathematical model to quantify whether HIV mutants with increased TIP resistance could stably invade high-risk populations. Strikingly, model results show that as long as TIP genomes are initially engineered to express at least ~3-fold more genomic RNA transcripts than HIV expresses in co-infected cells, TIPs can generally maintain population-scale stability. Further, due to two intrinsic evolutionary tradeoffs, TIPs are shown to be evolutionary stable at the population-scale whenever they are evolutionarily stable at the patient-scale.

## Methods

### Multi-scale model of HIV and TIP dynamics

**Fig 1:**
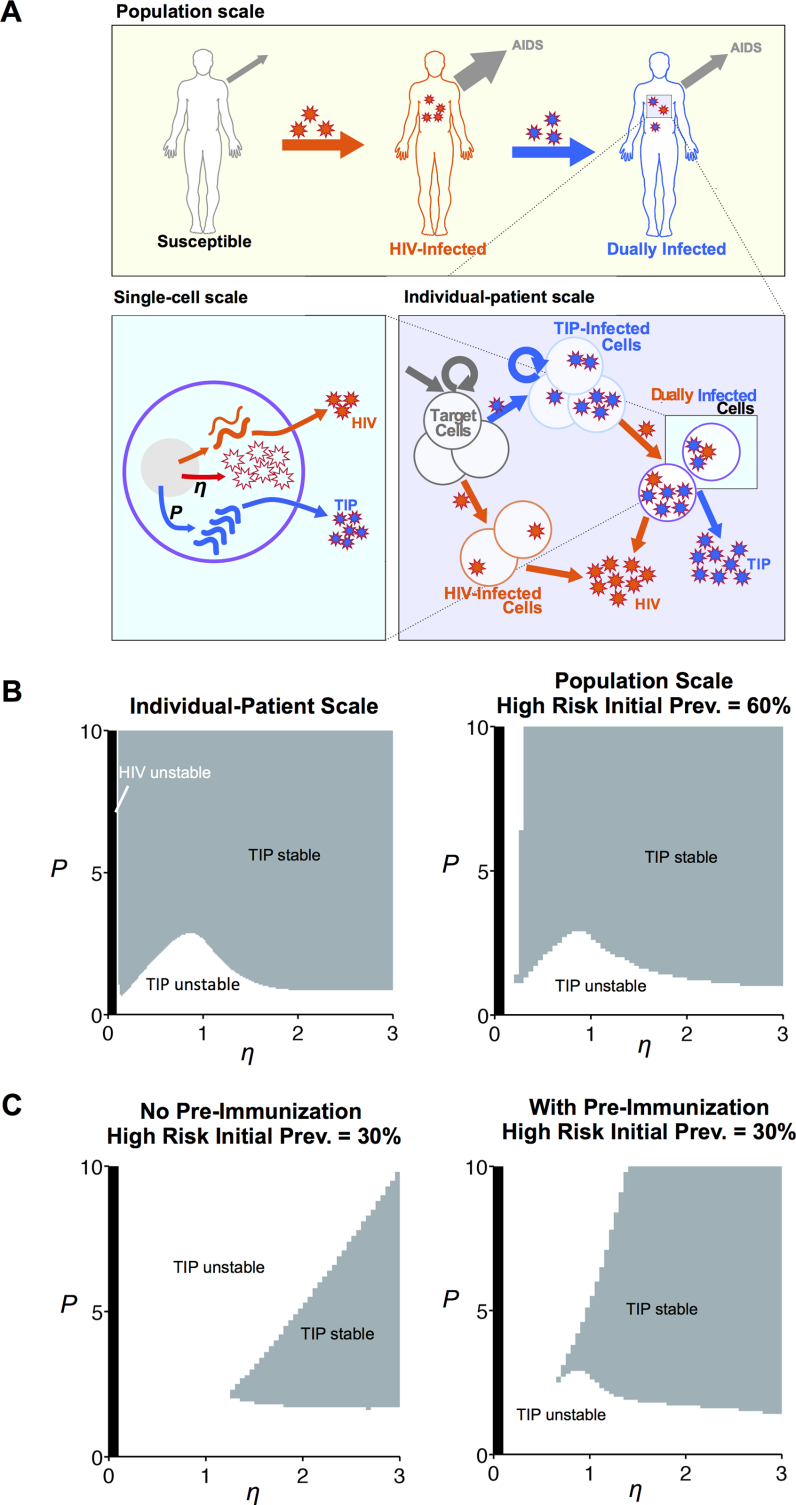
TIP and HIV are dynamically co-stable across multiple biological scales. **(A)** Schematic of the multi-scale mathematical model that tracks HIV and TIP levels across three biological scales: the single-cell scale, the individual-patient scale, and the population (i.e., epidemiological) scale. The cell-scale model quantifies TIP and HIV production in individual cells, as TIP and HIV genomic RNAs (gRNAs) compete for viral capsid proteins (i.e., public goods) produced by HIV (Section A in S1 Text). The design parameter *P* reflects the expression asymmetry between TIP and HIV gRNA expression; it quantifies the numeric advantage of TIP RNA genomes in acquiring HIV-produced viral capsids. The parameter *η* is intrinsic to HIV and quantifies the excess number of capsids relative to HIV genomes produced in HIV-infected cells. The outputs of the single-cell model (i.e., TIP and HIV burst sizes) are used as inputs for the patient-scale model. The patient-scale model is based on the standard model of viral dynamics [28] generalized to include TIPs and the division of CD4^+^ T cells (Section B in S1 Text). HIV infection prevents both cell division and super-infection of cells, while TIP infection permits cell division as well as super-infection by HIV and other TIP particles. The outputs of the patient-scale model (i.e., TIP and HIV viral loads) are used as inputs for the population-scale model. The population scale model is an epidemiological Susceptible-Infected (SI) model [27], again generalized to include TIPs (Eqs. 1 and Section C in S1 Text). For simplicity, only the key sub-population of high-risk disease spreaders is considered, as these individuals are responsible for a disproportionate fraction of HIV spread, including the spread of HIV resistance [24–26]. These high-risk individuals can be infected by HIV and can subsequently be superinfected by TIP to become dually infected. Death and transmission rates are calculated from HIV and TIP viral loads in hosts, following the approach validated in [22] (Section C in S1 Text). Panel adapted from [21]. **(B)** TIP invasion and dynamic stability in an individual host (left) and in a high-risk population (right) in which HIV’s prevalence is set at 60% prior to TIP introduction. TIPs are assumed to only infect hosts after a stable HIV infection has been initiated—i.e., there are assumed to be no (silent) TIP ‘pre-immunizations’ of hosts prior to HIV infection. TIPs engineered with *P* > 3 are stable except at low *η* values (near the region of HIV extinction). **(C)** TIP invasion and dynamic stability of a high-risk population in which HIV’s prevalence is only 30% prior to TIP introduction. The left panel quantifies TIP stability under the maximally conservative assumption that TIPs can only infect hosts after a stable HIV infection has been initiated, while the right panel quantifies TIP stability when TIPs are allowed to pre-immunize hosts prior to HIV infection. As the initial HIV prevalence increases, the TIP-stability regime at the population-scale approaches the TIP-stability regime within hosts.

The model of HIV and TIP replication and transmission includes three levels of biological organization: a population-scale model, an individual patient-scale model, and a cellular-scale model (Fig 1A). Each scale is represented by a well-studied system of deterministic ordinary differential equations. The population-scale model is an epidemiological Susceptible-Infected (SI) model [27] extended to include the spread of TIPs [18] (Section C in S1 Text). The patient-scale model is a variant of the basic model of HIV dynamics [28, 29], again extended to account for the presence of TIPs (Section B in S1 Text). Finally, the cellular scale model is a ‘public-goods game’—a well-studied system in game theory and evolutionary biology [30, 31]—in which HIV and TIP sequences compete for HIV capsid elements within dually-infected cells (Section A in S1 Text). Each scale’s equations are shown below, with all model parameters derived in the Supporting Information and summarized in Tables 1 and 2.

**Table 1:** Population-level parameters*.

Notation	Description	Units	Equation	Reference
*R*_0_^pop^	Basic reproductive ratio	*dimensionless*	Eqs S76, S78	
*μ*	Fold decrease in HIV transmission rate due to TIP presence	*dimensionless*	Eqs S82, S63, S62	[22]
*ϕ*	Ratio of TIP transmission to unsuppressed HIV transmission	*dimensionless*	Eqs S83, S64, S62	[22]
*B*	Lifespan decrease due to (unsuppressed) HIV infection	*dimensionless*	Eqs S77, S66, S68	[22]
*τ*	Lifespan increase due to superinfection (of HIV+ individual) by TIP	*dimensionless*	Eqs S84, S66, S67	[23]
*δ*_I_	Death rate of HIV^+^TIP^−^ individuals	years^-1^	Eqs S66	[22]

*With the exception of *δ*_I_, the above parameters are dimensionless, composite parameters that are used in the final population–scale model. For their definitions in terms of raw model parameters, and the full list of raw parameters, see S1 Text (Sec. A and B).

**Table 2:** Cell-level and host-level parameters*.

Notation	Description	Value	Reference
*Cell-level parameters*			
*κ*	Capsid and genome waste parameter	0.01	[19]
*P*	Ratio of TIP to HIV transcription rates	*free*	
*η*	Ratio of HIV capsid to genome production rates	*free*	
*Host-level parameters*			
*R*_00_ = *R*_0_^host^ (*η* = 1,κ = 0)	Within-host basic reproductive ratio	10	[66]
*δ/d*	Cell lifespan decrease due to HIV infection	10	[67]
*c*/*δ*	Ratio of viral to infected cell death rates	~10	[35]
*h*_0_	Maximum division rate of target cells per lifetime	3.3	[67]

*As in Table I, the above parameters are dimensionless. For their definitions in terms of raw model parameters, and the full list of raw parameters, see S1 Text (Sec. A and B).

At the single-cell scale, TIPs can only replicate and package by parasitizing essential *trans*-acting elements from full-length HIV in co-infected cells (Fig 1A, bottom left). In the absence of HIV, TIPs can only enter CD4^+^ T cells and integrate their genetic material into the cellular genomes—little to no TIP production occurs, since TIPs are engineered to lack essential *trans*-acting elements required for lentiviral replication and packaging. As a result, TIP genomic RNAs (gRNAs) only express after a cell is co-infected by HIV, at which point TIP gRNAs compete with HIV gRNAs for encapsidation by HIV capsid proteins. HIV capsids are thus intracellular ‘public goods’ that both HIV and TIP gRNAs utilize, as shown in the following equations:

$$\frac{dG_{\text{HIV}}}{dt}=\underset{\text{production}}{\underset{︸}{\theta }}-\underset{\text{packaging}}{\underset{︸}{k_{\text{pck}}G_{\text{HIV}}C}}-\underset{\text{decay}}{\underset{︸}{\alpha G_{\text{HIV}}}}$$

$$\frac{dG_{\text{TIP}}}{dt}=\underset{\text{production}}{\underset{︸}{mP\theta }}-\underset{\text{packaging}}{\underset{︸}{k_{\text{pck}}G_{\text{TIP}}C}}-\underset{\text{decay}}{\underset{︸}{\alpha G_{\text{TIP}}}}$$

$$\frac{dC}{dt}=\underset{\text{production}}{\underset{︸}{\eta \theta }}-\underset{\text{packaging}}{\underset{︸}{k_{\text{pck}}\left(G_{\text{HIV}}+G_{\text{TIP}}\right)C}}-\underset{\text{decay}}{\underset{︸}{\beta C}}$$

The three state variables represent the within-cell concentrations of HIV gRNA (*G*_HIV_), TIP gRNA (*G*_TIP_), and capsid proteins (C), respectively. As shown in the Supporting Information (Section A in S1 Text), the rate parameters *k*_pck_, *α*, and *β* can be grouped into a single composite waste parameter, *κ*, by non-dimensionalizing the model. The parameter *m* quantifies the number of TIP integrations, and results naturally from multiple TIP infections of a cell in the host scale model (below). The parameter *θ* is HIV’s gRNA production rate—*θ* serves to scale the size of all outputs, and is therefore absorbed into the host-scale parameters (Section B in S1 Text). Most importantly, *η* reflects the ratio of HIV-capsid to HIV-genome production and *P* corresponds to the ratio of TIP gRNA expression to HIV gRNA expression (i.e., the expression asymmetry). From previous studies, *η* and *P* are known to be two major parameters that determine TIP evolutionary stability within patients [18, 19]. Thus, we tracked TIP stability at the population scale as a function of *η* and *P*. For each value of (*η*, *P*), the single-cell scale equations are solved to determine HIV and TIP ‘burst sizes’ (i.e., the net numbers of HIV and TIP virions produced in the lifespan of an infected cell) (Eqs. 9–11 in S1 Text). The burst sizes are passed to the patient-scale equations, where they are used to calculate patient-wide viral set points, through the parameters *n*, *ψ*_m,_ and *ρ*_*m*_ (below).

At the patient scale, the model incorporates four fundamental asymmetries to determine TIP and HIV viral loads (Fig 1A, bottom right). (i) First, while TIPs require the presence of HIV to replicate, HIV can replicate in the absence of TIPs. (ii) Second, HIV gene expression (of the vpr gene) prevents the division of infected cells [32, 33]. Conversely, TIPs lack vpr and TIP infection is silent (absent HIV), so TIPs do not block cell division. (iii) Third, TIP-infected cells live as long as uninfected cells in the absence of HIV (due to this replicative silence); HIV infection results in rapid cell death [34, 35]. (iv) Fourth, HIV gene expression suppresses subsequent superinfection of a cell via the nef gene [33, 36]; TIPs lack nef and do not suppress superinfection. Thus, multiple copies of the TIP provirus can integrate into a cellular genome prior to HIV infecting that cell. These TIP infected cells can further divide to seed a reservoir of cells with a range of TIP proviruses. The resulting numbers of TIP and HIV particles in a patient are tracked in the following host-scale model:

$$\frac{dT_{0}}{dt}=\underset{\text{production}}{\underset{︸}{b}}+\underset{\text{division}}{\underset{︸}{dhT_{0}}}-\underset{\text{death}}{\underset{︸}{dT_{0}}}-\underset{\text{HIV infection}}{\underset{︸}{kV_{H}T_{0}}}-\underset{\text{TIP infection}}{\underset{︸}{kV_{T}T_{0}}}$$

$$\frac{dT_{m}}{dt}=\underset{\text{division}}{\underset{︸}{dhT_{m}}}-\underset{\text{death}}{\underset{︸}{dT_{m}}}-\underset{\text{HIV infection}}{\underset{︸}{kV_{H}T_{m}}}+\underset{\text{TIP infection}}{\underset{︸}{kV_{T}T_{m-1}-kV_{T}T_{m}}},\;m\geq 1$$

$$\frac{dI_{m}}{dt}=\underset{\text{HIV infection}}{\underset{︸}{kV_{H}T_{m}}}-\underset{\text{death}}{\underset{︸}{\delta I_{m}}}\;,\;m\geq 0$$

$$\frac{dV_{H}}{dt}=\underset{\text{HIV production}}{\underset{︸}{n\delta I_{0}+n\delta \sum_{m=1}^{\infty }\psi_{m}I_{m}}}-\underset{\text{clearance}}{\underset{︸}{cV_{H}}}$$

$$\frac{dV_{T}}{dt}=\underset{\text{TIP production}}{\underset{︸}{n\delta \sum_{m=1}^{\infty }\rho_{m}\psi_{m}I_{m}}}-\underset{\text{clearance}}{\underset{︸}{cV_{T}}}$$

The patient-scale state variables quantify: the numbers of HIV-uninfected cells with *m* TIP integrations (*T*_m,_ *m* ≥ 0), the numbers of HIV-infected cells with *m* TIP integrations (*I*_*m*_, *m* ≥ 0), the number of HIV virions (*V*_*H*_), and the number of TIP virions (*V*_*T*_). All host-scale parameters are described in the Supporting Information (Section B in S1 Text), where non-dimensionalization is shown to reduce the number of parameters to four: *R*_*0*_, *d*/*δ*, *c*/*δ*, and *h* (Table 2). Notably, HIV viral loads are lower in TIP-treated individuals than in HIV-only infected individuals, because cells co-infected with both HIV and TIP produce fewer HIV virions than HIV-only infected cells. The steady-state TIP (*V*_*T*_) and HIV viral loads (*V*_*H*_) resulting from this model are passed to a population-scale model to calculate virulence and spread across a population, as in [22].

The population-scale model (Eqs. 1 and Eqs. 59–61 in Section C in S1 Text) is a standard SI model with a single, well-mixed population, corresponding to high-risk disease spreaders [26]. As is common, a constant influx of susceptible individuals is assumed. These susceptible individuals are converted into HIV-infected individuals upon contact with an HIV-infected patient at a rate dependent on HIV’s viral load in the infected ‘donor’ patient [22]. While HIV can directly infect susceptible individuals, TIPs are (conservatively) assumed to only infect patients already infected with HIV. Further, co-transmission of HIV and TIP is neglected, due to the evidence showing that only a single founder virus generally establishes in patients after transmission through mucosal bottlenecks [37, 38]. Based on epidemiological and patient data [22], HIV infection progresses to AIDS as a function of HIV’s viral load in the patient, which reduces the effective lifetime of an infected individual and is modeled as removal from the population. Superinfection with TIP slows progression to AIDS (and reduces transmission of HIV from that individual) by reducing the HIV viral load (Fig 1A, top panel). The equations describing these epidemiological processes are:

$$\frac{dS}{dt}=\underset{\text{input}}{\underset{︸}{\lambda }}-\underset{\text{HIV infection}}{\underset{︸}{\frac{c}{N}\beta_{H}^{I}SI-\frac{c}{N}\beta_{H}^{I_{D}}SI_{D}}}-\underset{\text{death}}{\underset{︸}{\delta_{S}S}}$$

$$\frac{dI}{dt}=\underset{\text{HIV infection}}{\underset{︸}{\frac{c}{N}\beta_{H}^{I}SI+\frac{c}{N}\beta_{H}^{I_{D}}SI_{D}}}-\underset{\text{TIP infection}}{\underset{︸}{\frac{c}{N}\beta_{T}^{I_{D}}II_{D}}}-\underset{\text{death}}{\underset{︸}{\delta_{I}I}}$$

$$\frac{dI_{D}}{dt}=\underset{\text{TIP infection}}{\underset{︸}{\frac{c}{N}\beta_{T}^{I_{D}}II_{D}}}-\underset{\text{death}}{\underset{︸}{\delta_{D}I_{D}}}$$

The state variables represent the prevalences of: individuals susceptible to HIV (*S*), HIV-infected individuals (*I*), and dually infected (HIV^+^TIP^+^) individuals (*I*_*D*_). As shown in the Supporting Information (Section C in S1 Text), non-dimensionalization reduces the population-scale system to:

$$\begin{array}{c} \frac{dS}{dt}=\delta_{I}(\frac{1}{B}(1-S)-R_{0}^{\text{pop}}(SI+\;\mu SI_{D}))\\ \frac{dI}{dt}=\delta_{I}(R_{0}^{\text{pop}}(SI+\;\mu SI_{D}-\;\phi II_{D})-I)\\ \frac{dI_{D}}{dt}=\delta_{I}(R_{0}^{\text{pop}}\phi II_{D}-\tau I_{D}) \end{array}$$ (1)

The model parameters are defined as follows: *δ*_I_ is the rate of removal of HIV-infected individuals from the population (i.e. AIDS progression rate); *B* is the ratio of the removal rates of infected and uninfected individuals; *R*_0_^pop^ is the basic reproductive ratio of HIV in a population; *μ* and *ϕ* are the respective HIV and TIP transmission rates from TIP-treated individuals relative to the HIV transmission rate from individuals infected with HIV alone; *τ* is the decrease in the AIDS-progression rate due to superinfection with TIP. As in [22], these parameters are directly calculated from the HIV and TIP viral loads in the individual-patient model (Eqs. 62–69 in S1 Text and Table 1).

Following a well-established approach [19, 39, 40], the three biological scales (the single cell, the individual patient, and the host population) are integrated into a single multi-scale model, by using the steady-state outputs from the lower scale models as inputs into the higher scale models. This separation of timescales approach is possible, because the timescales of the processes that occur on each scale are so disparate that the processes on the lower scales are approximately at steady state relative to those on the higher scales. More specifically, the cell-scale processes reach steady-state in hours, the patient-scale processes require days to months, and the population-scale processes play out over decades.

Using this separation of timescales approach, the multi-scale model is simulated by setting values for *η* and *P* within single-cells. Inputting these values into the cell-scale model outputs HIV and TIP viral burst sizes. Using these burst sizes as inputs, the patient-scale model outputs set-point viral loads for HIV and TIP (Section B in S1 Text). Finally, the viral set-points are used as inputs into the population-scale model to calculate the parameters in Eq (1), specifically: the progression time of infected individuals to AIDS (*τ*) and the relative transmission rates of HIV (*μ*) and TIP (*ϕ*) (see [22, 23] and Section C in S1 Text). Thus, the final output of the multi-scale model is the prevalence of HIV and TIP across a population as a function of the intracellular design parameters *η* and *P*.

### Defining resistance at multiple scales

Once we can calculate HIV and TIP viral loads and prevalence levels as functions of *η* and *P*, we can then map the regions of the (*η*, *P*) parameter plane in which HIV mutants are able to maintain higher steady-state levels in the presence of TIP than is the wild-type HIV strain. We term these HIV mutants to be ‘resistant’ mutants. Given that the model covers multiple scales of behavior, there are multiple scales at which resistance can arise. At the host scale, HIV resistance corresponds to increased viral loads. Thus, HIV mutants that are able to maintain higher viral loads than wild-type HIV in the presence of TIP are termed ‘viral-load increasing’ resistant mutants. At the population scale, HIV resistance corresponds to an increased prevalence of unsuppressed (HIV+TIP-) individuals in the population. Thus, HIV mutants that are able to maintain a higher prevalence of HIV+TIP- hosts in the presence of TIP are termed ‘prevalence-increasing’ resistant mutants. Notably, at both host and population-scales, whether or not an HIV mutant is TIP-resistant is defined relative to the wild-type HIV strain—resistance is therefore dependent upon the parameter (*η*) values of both the wild-type and mutant HIV strains.

### Invasion analysis

After finding the parameter values that generate TIP-resistant viral phenotypes, the next step is to determine whether or not these resistant mutants can invade established populations of HIV and TIP to overcome a TIP intervention campaign. This invasion analysis is performed by taking the dominant eigenvalue of the Jacobian matrix of the system (Section D in S1 Text Eqs. 108–109), as is standard [41–43]. Behaviors at the host scale are independent of the population scale, so invasion within hosts can be solved agnostically of the population scale. On the other hand, whether or not a mutant can invade the population is dependent upon its behavior at the individual-host scale. To resolve this, we extend the model to allow for ‘host stealing’: take-over of hosts by the strain better able to propagate at the host scale. Because the time-scales differ greatly between the host and population scales, this host stealing is assumed to occur instantaneously from the perspective of the population. This ‘super-infection’ assumption is standard in multi-scale modeling studies in epidemiology [44]. Standard invasion analysis is then performed on the multi-scale model: a (TIP) treatment is termed ‘evolutionarily stable’ if no TIP-resistant HIV mutants are able to invade populations infected by wild-type HIV.

## Results

### Determinants of TIP dynamic stability at the population-scale

Using the multi-scale model described above, we first sought to determine the intracellular parameter regimes that result in TIP invasion and dynamic stability: i.e., the requirements for a small amount of TIP to invade a patient or population (*R*_0_^TIP^ > 1) and reach a stable nonzero steady-state. At the patient-scale, model simulations show that HIV can only achieve a nonzero steady-state when *η* > ~0.1, matching earlier predictions [19]. When *η* < ~0.1, HIV replicates so poorly within an individual that neither HIV nor TIP can propagate (Fig 1B, left and S1 Fig)—resulting in dual HIV and TIP extinction. When *η* ≈ 0.1, HIV achieves stability, and there is a small slice of the (*η*, *P*) parameter plane at which HIV is stable but TIP is not (Fig 1B, left). Everywhere else in the (*η*, *P*) parameter plane, TIP co-stability with HIV can be achieved with a sufficient level of TIP gRNA overexpression (*P*). In particular, when *P* > *P*_critical_ ≈ 3, TIPs are stable in the host across all *η* > ~0.1 and even remain stable as the TIP instability regime expands at *η* ≈ 1. Thus, engineering TIPs to express greater than ~3X more gRNA than HIV expresses is an essential design constraint at the patient-scale.

At the population-scale, *P >*~ 3 again generates TIP stability across a broad range of the (*η*, *P*) plane (Fig 1B, right). However, TIP stability also depends on the initial HIV prevalence in the high-risk population (in which HIV is highly prevalent) and on whether TIPs can ‘pre-immunize’ HIV-negative individuals (Fig 1C). For the maximally conservative assumption of no TIP pre-immunization, TIPs act as obligate secondary parasites and replicate only within HIV-infected individuals, so both the subpopulation available for TIP infection and the frequency of contacts within this subpopulation depend on the prevalence of HIV. Since the initial HIV prevalence depends on HIV’s basic reproduction ratio, $R_{0}^{\text{pop}}$ [45] (see Eqs. S78), the effective reproduction ratio of TIPs, *R*_eff_, is also dependent on the reproduction ratio of HIV within the population. The requirement for TIP spread and stability in a population under the conservative assumption of no TIP pre-immunization is (see Table 1 and Section C in S1 Text):

$$R_{\text{eff}}(\eta ,P\;)=R_{0}^{\text{pop}}-\frac{B\tau }{\phi }(\eta ,P\;)>1$$ (2)

Under this assumption, as the initial prevalence of HIV increases, the region of the (*η*, *P*) plane in which TIPs remain stable in the population expands and approaches the within-host stability region (Fig 1B). In particular, given a high-risk population with ~60% prevalence of HIV, if TIPs are stable in an individual, they stably coexist with HIV in the population. On the other hand, if pre-immunization is allowed and TIPs can (silently) pre-infect susceptible individuals and remain latent until subsequent infection with HIV (as was assumed in previous analyses [18]), the TIP stability region expands and is less sensitive to HIV’s initial prevalence (Fig 1C). Importantly, there is some evidence to suggest that pre-immunization is possible: lentiviruses are able to establish latency in rhesus monkeys even when suppressive antiretroviral therapy (which prevents replication) is started as little as three days post-infection [46]. Further, this latent state persists for more than six months [46]. In both cases, whether or not pre-immunization occurs, in regions of parameter space where TIPs coexist with HIV, TIPs will substantially suppress HIV/AIDS prevalence and incidence across a population (S2 Fig).

### Mapping regions of HIV resistance to TIP therapy

Having determined the critical engineering constraint for the dynamic stability of TIP treatments at both host and population scales—i.e., *P >* ~3—we next mapped the regions of parameter space at which HIV can achieve resistance to these stable TIPs. Since the model assumes no specific genotype-to-phenotype map, the difference between HIV strains is modeled phenotypically, as a difference in *η*. Importantly, the parameter *η* is likely to be under selection *in vivo*, as HIV has evolved suboptimal splicing (splicing increases *η*) and a molecular switch to control this splicing efficiency [47]. In contrast, the parameter *P* is assumed to be constant and independent of the HIV parameters (i.e., *P* depends on the TIP alone; see Discussion).

**Fig 2:**
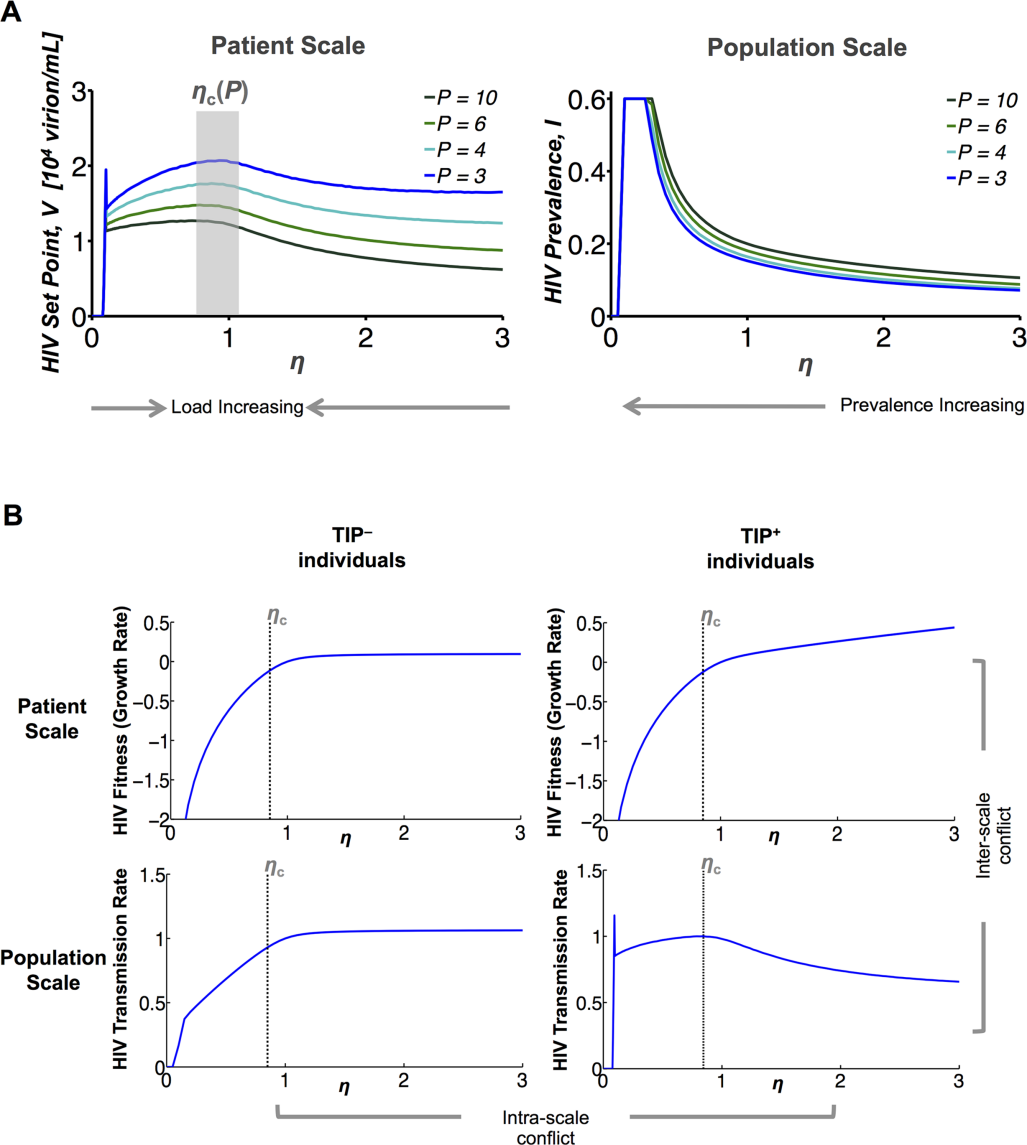
TIP-resistant HIV mutants face conflicting selection pressures. **(A)** Normalized HIV set-point viral load within individual patients (left) and steady-state HIV prevalence in a high-risk population (right) as functions of *P* and *η*. Only the TIP stability region of *P* > 3 is shown; S2 Fig shows the results when *P* < 3. For all *P* > 3, HIV’s set point peaks once at low *η ≈* 0.1 (near the HIV extinction regime), and again at a single critical value of *η* ≈ 1 termed *η*_c_(*P*) (translucent gray box). Since they increase HIV set points, both mutants with values of *η* closer to *η*_c_ (compared to wild type) or mutants close to ~0.1 are termed *virus-load increasing mutants*. In contrast, HIV prevalence always increases as *η* decreases. Mutants with lower values of *η* (compared to the wild-type) are thus termed *prevalence-increasing* mutants. Collectively, *virus-load increasing* mutants and *prevalence-increasing* mutants form the two classes of ‘resistant’ HIV mutants tracked in this study. **B)** Divergent selection pressures acting on *η* at the individual-patient scale (top panels) and the population scale (bottom panels). At the individual-level, relative fitness represents the relative rate of expansion or contraction of HIV mutants (via the effective selection coefficient; see Eq. 97 in S1 Text). At the population level, transmission rates are calculated from the individual patient viral set-point (see Eqs. 62–65 in S1 Text). Both fitness and transmission rates are normalized to their values at *η*_c_. In all plots, *P* = 6 (the features are qualitatively similar for all *P* > 3, S3 Fig). At the individual-patient scale, HIV fitness always increases as *η* increases—whether or not an individual has been exposed to TIP. However, in TIP-treated (TIP^+^) individuals, HIV’s population-scale transmission rate decreases as *η* increases above *η*_c_ (because increasing *η* also increases TIP production). The result is an *inter-scale conflict*, as divergent selection pressures at the individual-patient and population scales drive *η* in opposing directions in TIP^+^ individuals. Further, in TIP^−^individuals, HIV’s transmission rate increases whenever *η* increases. As a result, the presence of TIP^+^ and TIP^−^ individuals exerts opposing selection pressures on the value of *η*. We term this population-level conflict to be the *intra-scale* conflict.

As defined in the Methods (above), *η* mutants can generate two types of resistance relative to the wild-type *η* strain. HIV mutants that generate increased viral loads in an individual are considered TIP-resistant within the host, and are termed ‘viral-load increasing’ mutants. HIV mutants that generate increased prevalence in the population are considered TIP-resistant within the population, and are termed ‘prevalence increasing’ mutants. In TIP^+^ individuals, HIV viral loads reach a maximum at a critical value of *η* near *η* = 1 (Fig 2A, left). Since this maximal HIV viral load depends on the value of *P* (i.e., the particular TIP variant), we denote this critical value as *η*_c_(*P*). Any mutation in *η* toward *η*_c_ (i.e. any mutant for which |*η*_mut_ − *η*_c_| < |*η*_wt_ − *η*_c_|) is sufficient to increase the HIV load in TIP^+^ hosts (Fig 2A, left). Additionally, in a thin region of low *η ≈* 0.1, TIP is destabilized in HIV+ hosts, so HIV loads again peak. So, any mutant with *η ≈* 0.1 or with a value of *η* closer to *η*_c_ than the wild-type is a virus-load increasing mutant. In contrast, at the population-scale, mutants that reduce *η* reduce the population-level coverage of TIPs in the HIV-infected population, whatever the wild-type value of *η* (Fig 2A, right). Thus, any mutant with *η*_mut_ < *η*_wt_ is a prevalence-increasing mutant. Intuitively, both modes of TIP-resistance result from decreasing *η* to starve TIPs of the public goods (e.g., capsid proteins) they require. However, when *η* is decreased below *η*_c_ ≈ 1, decreasing capsid production begins to harm HIV’s own ability to propagate within hosts (so *η*_c_ ≈ 1 is effectively a ‘sweet-spot’).

Both viral-load increasing mutants and prevalence-increasing mutants can lead to either full or partial loss of HIV suppression, and, consequently, full or partial elimination of TIPs from the pertinent scale (Fig 2A and S2 Fig). Full resistance at each scale (individual or population) essentially drives the system to regions of *P* and *η* where TIPs are unstable (Fig 1B and 1C and S2 Fig). However, if *P* > 3, full resistance to TIP treatment only occurs at low *η* values of ~0.1, near the HIV extinction threshold (Fig 2A).

### HIV escape mutants face conflicting selection pressures

After mapping the regions in which HIV escape mutants arise, the next step is to whether or not these resistant mutants can spread across a population to undermine a TIP campaign. To do so, we examine the introduction of mutant HIV strains into the host population and analyze the competition between the wild-type HIV strain and each new mutant HIV strain. HIV mutants are introduced in small quantities into an individual with steady-state TIP and wild-type HIV viral loads. As in the stability analysis above, we rely on a time-scales separation, since a large number of viral replication events (i.e., viral generations) occur within each individual between inter-individual transmission events. Examining the dominant eigenvalues of the Jacobian, we determined the fitness landscape for HIV mutants at the individual-patient level from the rate of expansion or contraction of a mutant strain with slight differences in *η* relative to the wild-type (Section D in S1 Text). The Jacobian analysis enables us to calculate the net selective advantage (or disadvantage) of any TIP-resistant mutants.

There are two scales at which evolutionary fitness must be analyzed: the host scale and the population scale. At the host scale, the relative fitness of an HIV variant reflects the relative growth rate of that clone within a host. For any HIV strain, growth rate increases with its effective reproductive ratio [19], which depends on the viral burst sizes from individual cells and the distribution of TIP multiplicities among the cells. Critically, within a given cell, a larger *η* always corresponds to a larger viral burst size, regardless of the TIP multiplicity (S1 Text Eqs. S9-S13). Consequently, HIV mutants with larger values of *η* always have higher relative fitness within an individual, regardless of the presence or level of TIP (Fig 2B, left; and S3 Fig). This result can be understood intuitively as a ‘tragedy of the commons’ [48, 49]: enhanced capsid production favors the HIV strain that can achieve it, despite enabling increased TIP parasitism of all HIV mutants in the host.

At the population scale, the effective reproductive ratio of HIV is determined by its ability to transmit between members of the host population [22]. The HIV transmission rates are calculated (Eq 1, Table 1) from the viral load outputs from the individual-patient model as in [18] (see Section C in S1 Text). Since these patient-level viral loads themselves depend on the single-cell parameters, the transmission rates of HIV mutants are ultimately functions of *η* and *P*. Further, these transmission rates differ greatly between individuals only infected with HIV alone individuals infected with both HIV and TIP. In the absence of TIP, more resource production (i.e., a higher *η*) is always better for HIV, so HIV transmission always increases with *η* in TIP^−^ individuals (Fig 2B, top right). However, in TIP^+^ individuals, the transmission rate and the viral load both peak at *η* = *η*_c_ (Fig 2B, bottom right). Intuitively, in TIP^+^ hosts, there is a balance between producing enough resources to propagate, and producing too many resources, which allows TIPs to establish a larger population. Taken together, the model results capture conflicting selection pressures driving HIV transmission from TIP^+^ individuals, HIV transmission from TIP^−^ individuals, and HIV viral loads within patients. These conflicting pressures push HIV evolution in different directions along the *η*-axis, resulting in evolutionary conflicts on the value of *η*.

Overall, two evolutionary tradeoffs emerge from the model: an *inter-scale* conflict in TIP co-infected individuals between host-level HIV fitness and population-level HIV transmission, and an *intra-scale* (population-level) conflict between HIV transmission from individuals co-infected with TIP and individuals not co-infected with TIP (Fig 2B). The *inter-scale* conflict arises from the fact that when TIP is evolutionarily stable on the host level, evolution within TIP-treated hosts leads to higher *η* values. Yet, at the population-level (i.e., in TIP+ individuals), HIV variants with lower *η* values are evolutionarily beneficial, since they reduce TIP levels and attendant parasitism. The *intra-scale* conflict also arises from the benefit to HIV of reducing *η* in TIP^+^ populations—the tradeoff is that in TIP^−^ populations, reducing *η* actually reduces HIV loads and transmission. Thus, the intra-scale conflict exists within the population scale alone and is dependent on the frequency of TIP^+^ hosts (i.e., it is a frequency-dependent effect).

### Evolutionary conflicts prevent the establishment of resistant HIV mutants

**Fig 3:**
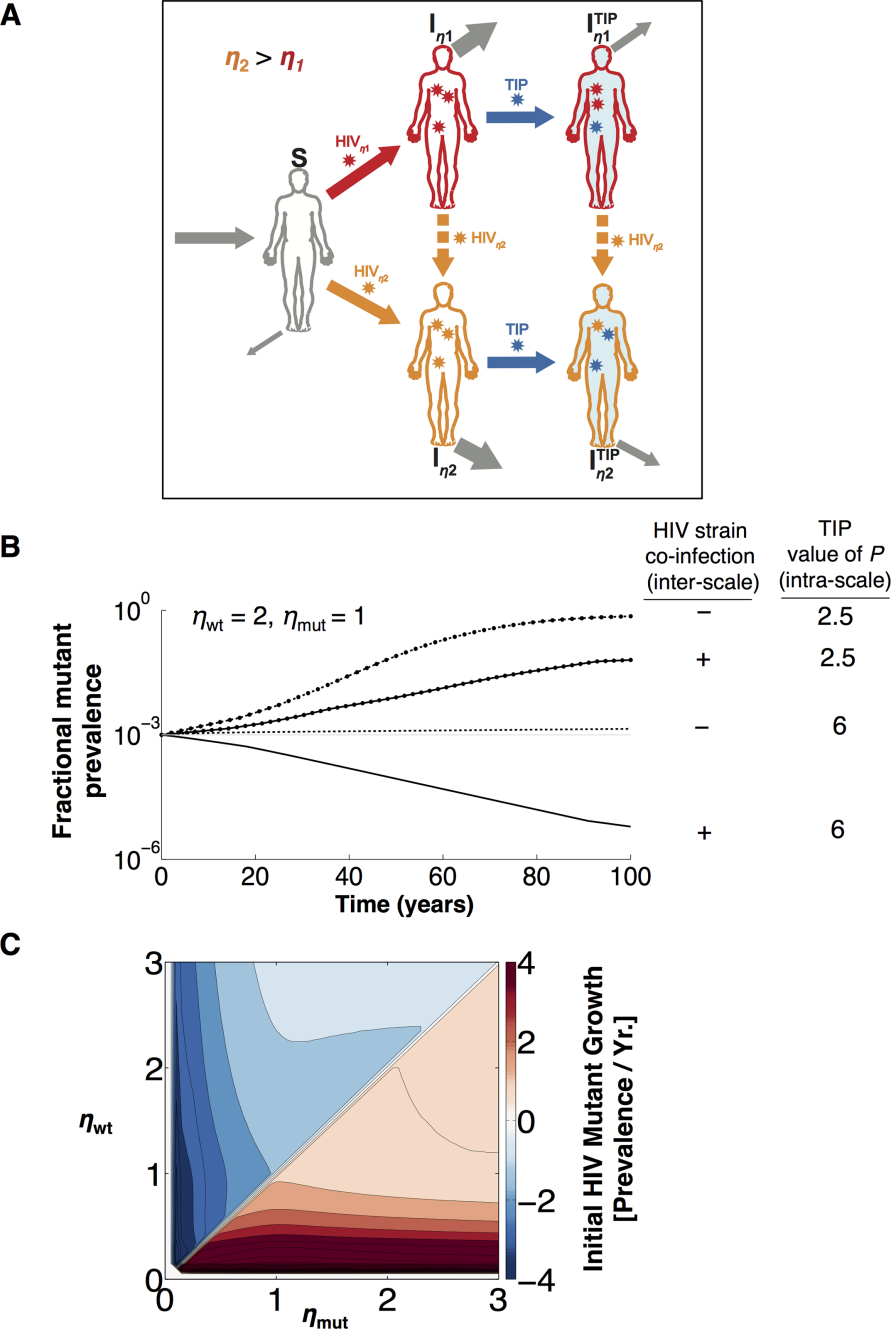
Conflicting selection pressures prevent the outgrowth of TIP-resistant HIV mutants in a host population. **(A)** Schematic of extended model built to examine the outcome of the evolutionary conflicts. Two HIV strains with different *η* values compete for transmission across a TIP-exposed population (Eqs. 100–104 in S1 Text). When multiple HIV strains co-infect the same individual, the HIV strain with highest *η* outcompetes all other HIV strains due to its higher replicative fitness (see Fig 2B); this host-level outcome occurs rapidly compared to dynamics on the population-scale (enabling a time-scale separation). **(B)** Projected spread of HIV mutants with reduced *η* values across a TIP-treated HIV population. The wild-type HIV strain has *η*_wt_ = 2 and the mutant HIV strain has *η*_mut_ = 1. The inter-scale evolutionary conflict is only present when co-infection of an individual host (by both wild-type and mutant HIV strains) is allowed. Co-infection enables host-level selection against the mutant HIV strains with decreased values of *η*, because of their decreased replicative fitness (despite their population-level transmission advantage). The intra-scale conflict is only significant when *P* > 3. For example, at *P* = 2.5, decreasing *η* results in major increases in transmission from TIP^+^ individuals (Fig 1B) that overwhelm the modest decreases in transmission from TIP^-^ individuals, essentially removing the evolutionary conflict at the population-scale. In contrast, at *P* = 6, decreasing *η* causes comparable and opposing changes in transmission rates from TIP^+^ and TIP^−^ individuals (Fig 2B). Overall, the presence of either co-infection or TIP stability (*P* > 3) is sufficient to decrease the level of mutant spread, but in the presence of both conflicts, HIV mutants with reduced *η* are completely prevented from spreading. **(C)** Pairwise invasability plot showing the initial expansion or contraction rate of a mutant HIV strain (*η*_mut_) upon introduction into a population infected with both TIP and wild-type HIV (*η*_wt_). Each HIV mutant and HIV wild-type combination is represented by an (*η*_mut,_ *η*_wt_) point on the plane. At each point, the color of the heatmap shows the maximal expansion rate (i.e., eigenvalue of the Jacobian) for the mutant strain, across all *P* > 3. The maximal eigenvalue represents the worst-case scenario, when the mutant spreads most easily. When *P* > 3, the only mutants that can grow are those with *η*_mut_ > *η*_wt_. Thus, HIV appears unable to unilaterally mutate away from TIP control by reducing its production of *trans* elements (i.e., starving the TIP), when TIPs are engineered with *P* > 3.

Given these two evolutionary conflicts on the value of *η*, we probed which HIV mutants could spread through a population by extending the population-level model to include multiple HIV strains (Fig 3A and S1 Text Eqs. 100–104). For each HIV strain, relative transmission rates were calculated based on viral loads, as in the one-strain model (Eq 1, Table 1). When wild-type and mutant HIV strains co-infect the same host, host fitness comes into play at the population scale, since the within-host infection dynamics occur extremely rapidly relative to the population-scale dynamics [50]. As a result, the more fit HIV strain takes over within a host prior to population-scale transmission events. The rapid host take-over is due to ‘competitive exclusion,’ which precludes two strains from coexisting at steady state, regardless of the presence of TIP (dashed vertical arrows in Fig 3A). Therefore, we neglect individuals co-infected with multiple HIV strains—the most fit HIV strain rapidly excludes the others (see the Discussion for an analysis of cases where competitive exclusion does not occur, due to weakened within-host selection).

The dual-strain model was first used to track whether a representative HIV mutant with *η*_mut_ = 1 can invade a TIP-treated population with a wild-type HIV strain in which *η*_wt_ = 2 (Fig 3B). Given its decreased *η* value, this HIV mutant would increase the HIV viral load in HIV+TIP+ (co-infected) individuals and increase the prevalence of HIV+TIP- individuals in a population (Fig 2B). However, the mutant would be disfavored in TIP^−^ populations and disfavored within individual hosts. The dual-strain model weighs the conflicting selective benefits at both scales to calculate whether the HIV mutant can spread.

In the particular example of *η*_wt_ = 2 and *η*_mut_ = 1, the dual-strain model demonstrates that the overall population-level trajectory of the resistant HIV mutant is toward extinction—given both large *P* (i.e., *P > 3*) and the presence of co-infection (Fig 3B and S4 Fig). Effectively, *P* > 3 drowns out the selective advantage of decreasing *η*: the modest increases in transmission from TIP^+^ individuals are matched by decreases in transmission from TIP^-^ individuals. In contrast, when *P* = 2.5, decreasing *η* results in a major increase in transmission from TIP^+^ individuals (Fig 1B), which dwarfs the decrease in transmission from TIP^-^ individuals. Thus, increasing *P* to a value greater than 3 is required for a robust *intra-scale conflict*. The trajectory of the mutant strain also depends on whether co-infection can occur, since co-infection results in the out-competition of reduced *η* mutants within hosts despite their population-level fitness advantage. In other words, co-infection is required for an *inter-scale conflict*. Competing mutant and wild-type HIV strains in the presence and absence of *P > 3* and co-infection demonstrates that both intra-scale and inter-scale conflicts are necessary to prevent the establishment of TIP resistance (Fig 3B).

To determine the general conditions across all of (*η*, *P* > 3) parameter space under which a mutant virus (with parameter *η*_mut_) can spread into a wild-type-infected host population (with parameter *η*_wt_), we performed an invasion analysis [41–43] to examine the initial expansion rates of HIV mutants after introduction (Fig 3C). When *η*_mut_ *< η*_wt_, the model shows that HIV mutants never expand (Fig 3C). This result is the key to determining parameter regions of *P* where TIPs would be safe from HIV escape mutants (i.e. the design criteria for engineering ‘resistance-proof’ TIPs). Indeed, if the small HIV-mutant population shrinks initially, it will never be able to outcompete wild-type HIV. Thus, when *P* is safely in the TIP stability region of *P* > ~3, HIV evolution is constrained to move toward larger *η* values and away from TIP-resistance.

In terms of the two types of resistant mutants discussed above, this invasion analysis (Fig 3C) shows the extinction of *prevalence-increasing* mutants anywhere, and the extinction of *virus-load increasing* mutants when *η*_wt_ *> η*_c_. *Virus*-*load increasing* mutants do spread in a host and in a population when *η*_wt_ *< η*_c_, in which case all the selection pressures align (S5 Fig), because an increase in *η* results in an HIV load increase. However, in this range (*η*_*wt*_ *< η*_*c*_), HIV is pressured toward higher *η* values regardless of the presence of TIP (Fig 2B). This selection pressure arises not from the presence of TIP, but from the enhanced replication of HIV at the host scale at higher capsid production rates (regardless of TIP). At the population level, the selection pressure towards higher *η* even decreases when TIP is present (see the transmission rates in Fig 2B, right). In other words, the population-level instability comes from a pre-existing host-level instability, prior to the introduction of TIP.

Taken together, the results of the invasion analysis show that—given dynamic and evolutionary stability at the host scale (i.e., *P* > 3 and *η*_wt_ *> η*_c_)—TIP interventions would be both dynamically and evolutionarily stable from unilateral HIV escape mutants at the population scale.

### Robustness of results to model assumptions

While the models used to test TIP evolutionary stability at both host and population-scales are well-established [45], as in any modeling study, our analysis necessarily utilizes simplifying assumptions. To determine whether these simplifying assumptions impacted model outcomes, we performed a number of sensitivity analyses in which model assumptions were relaxed. For example, a concern in the host-scale model is the function used to model target-cell division (since cell division enables the vertical transmission of provirally integrated TIPs across a host). To keep the uninfected T-cell population bounded, we assumed that the cell division rate ‘shuts off’ at high T-cell concentrations. Yet, the form of the function used to model this homeostatic shutdown could, in theory, affect the model’s outcomes. We thus tested disparate shutdown functions, finding that large changes in the shutdown function only result in small changes in the dependence of the equilibrium target-cell division rate (h_eq_) on the maximal target-cell division rate (h_0_) (S7 Fig). This is because the equilibrium target-cell division rate is mostly driven by the asymptotic T-cell level, which is independent of the form of the shutdown function (SI Section B).

A second assumption is that all TIP-immunized hosts have significantly reduced HIV transmission rates, due to a TIP-mediated reduction in HIV viral loads. However, a large fraction of HIV transmission can occur prior to TIP suppression, especially during the acute phase of infection [51–53]. To account for the possibility that a large fraction of HIV transmission by a patient occurs during the acute phase of infection prior to TIP suppression of viral loads, we re-analyzed the model under the strong assumption that TIP therapy does not reduce HIV transmission at all in dually-infected (i.e., TIP^+^, HIV^+^) individuals. This is equivalent to the (worst-case) assumption that all of HIV’s spread occurs during acute infection prior to TIP inoculation. Importantly, the results of the model are virtually unchanged—both TIP dynamic stability (S8A Fig) and TIP evolutionary stability (S8B Fig) are preserved. Intuitively, the reason for the sustained TIP efficacy despite high HIV transmission is that increased HIV transmission enables increased TIP colonization of the population (*middle* columns of S8 Fig). Thus, the increased HIV levels only strengthen the evolutionary stability of TIPs to HIV mutants (*last* columns of S8 Fig).

Finally, we examined whether increased death rates reduce the transmission potential of higher *η* (i.e., TIP-susceptible) mutants and thereby select for lower *η* (i.e., TIP-resistant) strains. In fact, the increase in death rates due to increasing *η* only has a minimal effect on the transmission potential (S9 Fig). This robustness occurs because the increase in viral loads saturates as *η* is increased. Further, this saturation point is at a viral set-point of ~10^5^, whereas the measured sharp decrease in transmission potential occurs at a set point >10^5^ [22].

Overall, the results of these sensitivity analyses support the earlier model results, showing that TIPs can be engineered to be both dynamically and evolutionarily stable at the population-scale.

## Discussion

Here we developed a three-scale model of HIV dynamics to test whether interfering particles—which parasitize critical HIV proteins within individual cells—can be designed to stably control the rapidly evolving HIV virus throughout a high-risk population. In the absence of HIV mutation, the analyses show that TIP interventions can spread and stably persist at the population-scale, whenever there is sufficient prevalence of HIV within the high-risk population (Fig 1B). Importantly, even if HIV’s prevalence is initially low in the high-risk population, TIPs remain *dynamically* stable if they can pre-immunize HIV^-^ individuals (Fig 1C). The analyses further tested the *evolutionary* stability of TIP interventions at the population-scale, probing whether ‘resistant’ HIV mutants that lead to increased HIV viral loads within patients or increased HIV prevalence across populations can undermine TIP efficacy. Critically, model results show that the spread of these TIP-resistant HIV mutants is limited by two fundamental evolutionary tradeoffs: their reduced transmission rates in untreated individuals and their reduced growth rates in both untreated and treated individuals (Fig 2B). In fact, when TIPs are evolutionarily stable within hosts, these evolutionary conflicts drive HIV to evolve toward increased (rather than decreased) TIP susceptibility at the population-scale (Fig 3C). Taken together, the analyses show that whenever TIPs can be designed to be dynamically and evolutionarily stable in individual patients, they remain dynamically and evolutionarily stable in populations.

### Precedents for multi-scale analysis

The competition of mutant pathogen strains across multiple biological scales has previously been considered in a number of studies, as reviewed in [43]. Notably, these studies often predicted that co-infection would lead to increased pathogen virulence, because more virulent strains are likely to replicate more rapidly (i.e., have increased fitness) [49, 54, 55]. This increased virulence result is, in many ways, analogous to our finding that HIV always evolves toward higher *η*—except that increased *η* in the context of TIP therapy results in *decreased* HIV virulence. Importantly, *decreased* virulence is a predicted outcome in ‘public goods’ models in which selfish, but less virulent pathogens outcompete cooperative, virulent strains [31]. In the public goods framework at the host-scale, TIPs are the public goods shared among the HIV strains co-infecting a host. Critically, an individual HIV strain benefits when there is *more* TIP production, because increased capsid production increases both TIP production and that strain’s relative fitness. Yet, increased TIP production is deleterious to the overall HIV population, reducing all HIV strains uniformly. Thus, evolution toward increased TIP production can be viewed as evolution toward a cheating HIV strain, explaining the overall virulence reduction as a viral ‘tragedy of the commons’ [48, 49].

Still, a key question of this study was to determine whether the cheating HIV strain would outcompete cooperative HIV strains that produce fewer TIPs, since TIP production decreases HIV transmissibility at the population-scale. As analyzed in detail in [39, 56], whether or not a cheater outcompetes a cooperator in a multi-level evolutionary conflict depends on the relative strength of the within-host evolutionary pressures (which favor the cheater) and the between-host evolutionary pressures (which favor the cooperator). Our results capture the dominance of the within-host pressures in the context of HIV-TIP dynamics, because the between-host pressures in favor of decreased TIP production are absent (and in fact inverted) in the sub-population that remains TIP^-^.

### Model assumptions and limitations

In the multi-mutant model, new HIV mutants are assumed to arise infrequently relative to the strength of within-host selection—i.e., a weak-mutation, strong-selection regime is assumed. In fact, the multi-mutant model assumes an extreme weak-mutation regime, with HIV mutants only introduced into patients via co-infection. This neglects the *de novo* generation of new HIV mutants within hosts, which, in truth, occurs rapidly for an RNA-encoded virus. Fortunately, this minimization of HIV mutation represents a worst-case scenario for demonstrating TIP evolutionary stability at the population-scale. If within-host mutations were to arise more frequently, the effects of host-level selection would only become more pronounced at the population scale. This is because there would be greater numbers of cheater HIV mutants that increase TIP production (i.e., HIV mutants with higher *η* values than the wild-type). Further, all TIP-resistant mutants with lower *η* values than the wild-type would be lost due to their selective disadvantage within hosts. The increased mutation of HIV strains would thus enable the emergence of HIV mutants with higher *η* values, enhancing the evolutionary stability of TIP treatments. Consequently, once evolutionary stability has been established in a multi-mutant model in which host-scale effects are only exerted through co-infection, stability in a model with *de novo* generation of mutants follows.

In addition to changing the strength of mutation, one could also study how the results hinge on the strength of selection. The assumption that TIP-susceptible (i.e., higher *η*) HIV strains competitively exclude TIP-resistant (i.e., lower *η*) HIV strains within hosts depends on the strength of within-host selection. If host-scale selection is too weak, a distribution of mutants with different *η* values (i.e., TIP resistance levels) could persist within hosts. However, the presence of a distribution of mutants (rather than a single mutant) does not obviate either TIP dynamic or evolutionary stability. We begin by considering TIP dynamic stability in the context of decreased within-host selection. Given that HIV’s burst size increases monotonically in *η*, any within-host distribution of *η* mutants can be modeled as a single ‘characteristic' *η* mutant: the ‘characteristic’ HIV mutant whose *η* value gives rise to the average HIV burst size in the host. As long as the *η* value of this mutant is within the stability regime derived in Fig 1B for the single-mutant case (i.e., *η* > ~ 0.2), TIPs will remain dynamically stable at the host and population scales. For evolutionary stability, the key point is that TIP introduction leaves any distribution of *η* mutants essentially unchanged (if anything, it shifts the distribution slightly toward higher *η*: Fig 2B). This is because TIPs suppress all HIV mutants within a host essentially uniformly (they all feel the same TIP load). Thus, both the relative fitness values (Fig 2B) and relative transmission rates of the HIV mutants are unchanged by TIP introduction. As a result, there is no change in the patient-scale *or* population-scale distribution of *η* mutants and no selection for TIP-resistant strains. These arguments aside, if the strength of mutation were significantly increased, it might be possible for a beneficial mutant to arise that happens to have both a lower *η* and a net fitness advantage due to secondary beneficial mutations. We neglected these higher-order effects due to factors such as genetic linkage and clonal interference—as well as non-deterministic effects due to factors such as Muller’s ratchet and genetic drift—in our simplified model. Following a number of recent studies in theoretical population genetics [50, 57–59], these ideas could be the subject of future models.

A more basic assumption of the model is that HIV escapes TIP parasitism by reducing *η*—*i*.*e*., by unilaterally reducing the production of capsid elements available for TIP parasitism. Since TIPs do not encode *trans* elements such as capsids, the TIPs would have no ability to restore *η* to high values (*i*.*e*., *η* is an *asymmetric* parameter). Consequently, HIV’s *unilateral* evolution of *η* offers the most direct route for HIV to evade TIP-mediated suppression. Yet, as an alternative to this unilateral evolution in *η*, one could consider mutations in *θ*, which would alter HIV’s gRNA production rate.

Importantly, mutations in *θ* would be more *symmetric*, changing the production rates of both HIV and TIP. For example, by increasing HIV’s genomic RNA (gRNA) production rate, increasing *θ* would also increase the production of the HIV protein (Tat) that transactivates the LTR promoters of both HIV and TIP and increase the production of the HIV protein (Rev) that exports both HIV and TIP gRNAs into the cytoplasm for encapsidation. Thus, TIP gRNA production would be increased in symmetry with HIV gRNA production. A key point is that if the TIP:HIV gRNA overexpression ratio (*i*.*e*., *P*) were still sufficiently high—i.e., if *P* were still *>3*—the TIPs’ evolutionary stability would be expected to remain.

A further reason that HIV mutations in *θ* are unlikely to generate stable TIP-resistance is that increasing *θ* may not be an evolutionarily stable strategy for HIV—an intrinsic fitness cost may prevent HIV from increasing *θ*. In particular, a recent study [61] showed that increasing the rate of transcription (e.g., by adding transcription factor binding sites to the LTR) reduces the level of HIV replication, likely by disrupting an evolutionarily-tuned viral replication program. If *θ* has been optimized over the millennia of lentiviral evolution in primates, then a similar cross-scale evolutionary conflict to the one shown here for *η* could limit the emergence of HIV mutants with increased *θ* values, despite their increased TIP-resistance potential.

Conversely, if increasing *θ* were evolutionarily beneficial to lentiviruses, then TIPs could directly co-evolve to match HIV evolution for a ‘symmetric’ parameter such as *θ*. This is because, unlike *trans* (e.g., capsid) elements, TIPs encode all *cis*-acting elements. And given their shared error-prone reverse transcriptase enzyme, TIPs have the same evolutionary capacity and pressure to modify their LTR promoter towards increased gRNA production, should increased gRNA production prove beneficial within a cell. Thus, ‘red queen’ type selection races may arise [60], with both TIP and HIV particles simultaneously adapting to attempt to gain the upper hand in gRNA production (i.e., with both TIP and HIV evolving to modulate the level of gRNA overexpression, *P*). In fact, one numerical simulation study appears to have demonstrated this [20], although the particular methodologies and assumptions have been questioned [62, 63]. Taken together, given the potential of TIPs to co-evolve in *θ* and the simpler possibility that *θ* is already evolutionarily optimized, this study assumes that *θ* remains fixed, as in previous studies [12, 18, 19]. With *θ* fixed, *P* is similarly fixed once the TIP has been engineered.

A final non-unilateral escape mechanism involves mutations in the HIV *trans* elements that the TIP parasitizes. It has been argued that HIV is unlikely to win the resulting *cis-trans* arms races with TIP, due to an intrinsic mutational asymmetry between HIV and TIP [18, 19]. To escape TIP parasitism, HIV needs to almost simultaneously adapt both its *cis* and *trans* elements in a correlated way, so that the mutant *trans* still interacts with the mutant *cis* but not the original *cis* element (which remains in the TIP). Within this same adaptation timeframe, the TIP only needs to mutate its corresponding *cis* element to keep pace. Since both HIV and TIP maintain the same mutation rate—TIPs share the same error-prone reverse transcriptase protein that drives HIV mutations—and TIPs need to mutate fewer elements, TIPs would have a built-in evolutionary advantage. Notwithstanding this advantage, the outcomes of these arms races may depend on other factors, such as the ability of an individual TIP to parasitize distinct HIV mutants. Thus, detailed simulations of arms races between HIV and TIP will be carried out in a subsequent study.

### Caveats to modeling multi-level selection

As a result of the conflicting selection pressures induced by TIP, there are important caveats for modeling the evolutionary behavior of HIV in the presence of TIP. In general, the selective forces at the population level cannot be described by a fitness landscape. In order to express selection acting on a mutant as a fitness landscape, it must be possible to express the relative slope of expansion, *s*, as the difference in log fitness (*f*) between two strains, *s* = *f*(*η*_1_, *η*_2_) = *f*(*η*_2_) − *f*(*η*_1_), where *η*_1_ and *η*_2_ are initial and final values, respectively. However, this condition is violated in the present model for three reasons: (i) in the case of co-infection with different HIV strains (leading to competitive exclusion), the less host-fit mutant experiences a negative-selection pressure at the population level with the strength of this pressure depending on the frequency of contacts with individuals infected with fitter strains; (ii) the initial expansion rate of mutant strains has a term that does not depend on the magnitude of the parameter difference from the wild type (*η*_1_ − *η*_2_) but only on its sign, because of the speed of within-host competitive-exclusion relative to the population time-scale; (iii) even in the absence of within-host co-infection, the spread of a strain depends on the balance between TIP^+^ and TIP^−^ individuals in the population and this ratio adjusts with the prevalence of the strains, causing long-term oscillations (S6 Fig).

Given the importance of the intra-scale (i.e., population-scale) conflict and the resultant frequency-dependent fitness effects at the population scale, it could be reasonably expected that similar features would appear at the host-scale. However, these frequency-dependent features can be safely neglected in this model, where we calculated a fitness landscape corresponding to incremental small changes in *η* (Fig 2B). Although modest frequency-dependent corrections would appear for large jumps in parameter *η*, these effects only adjust the strength of selection, not the direction. Because we assumed a strong separation of timescales between the host and population levels, only changes in the direction (not the magnitude) of host-level selection affect the final results. Within hosts, HIV fitness always increases as *η* increases regardless of how many TIP copies are in a cell (i.e., regardless of TIP and HIV loads in a host), since a larger *η* always results in a larger burst size (S1 Text Eqs. S9-S13). Hence, regardless of TIP frequency, the HIV strain with largest *η* always spreads fastest in a host, driving the other strains to extinction.

### Design considerations for resistance-proof therapies

The predicted lack of unilateral evolution of HIV towards resistance to TIP is in striking contrast to HIV resistance to antivirals, which commonly arises in treated individuals due to poor adherence or suboptimal therapy regimens [64, 65]. HIV strains that are resistant to antivirals can then transmit from host to host, spreading through the population [65]. The critical difference between antivirals and TIPs is that TIPs parasitize HIV *trans* elements (i.e., steal HIV proteins), and this parasitism inexorably uses the same biochemical processes as HIV replication. So, to prevent TIPs from interfering, HIV must interfere with its own ability to replicate (i.e., ‘shoot itself in the foot’). In other words, the cost of mutation is always directly related to the benefit of the mutation. In contrast, HIV-escape from antiviral pharmaceuticals may produce some disadvantages for the mutant strain relative to the wild-type strain, but the benefit of the mutation and the cost of the mutation are not necessarily related.

With each TIP-evading mutation necessarily arising at a cost, our analysis quantifies the net-benefit (i.e., evolutionary viability) of these mutations across a population, where resistance may be beneficial to transmission. By capturing the parameter regimes under which resistance mutations are driven extinct, the analysis offers general guidelines for engineering therapies that obviate the spread of antiviral resistance within populations; in fact, these therapies are likely to direct pathogens toward increased susceptibility. As a result, these design constraints may aid in the engineering of resistance-proof interventions against a range of viral and bacterial pathogens beyond HIV.

## Supporting Information

S1 Text Detailed analyses of mathematical models. Derivations and non-dimensionalizations of the cell-scale, patient-scale and population-scale mathematical models. TIP invasion and dynamic stability conditions are derived at each scale, as is the condition for the evolutionary stability of the TIP to (unilateral) HIV mutations in *η*. Further, sensitivity analyses are performed to test model robustness.
(PDF)

S1 Fig TIP intervention suppresses HIV viral loads within a host. (A,B) Set-point levels of TIP and HIV within a host, measured in units of viremia (RNA copies/ml). The orange region in the X-Y plane denotes the region of within-host TIP instability. The average HIV viral load prior to TIP intervention is assumed to be 10^5^. Model parameters are as listed in S1 Table.
(PDF)

S2 Fig HIV loads in a host and HIV prevalence in a population. Heatmap analogs of Fig 2A expanded to include the region when *P* < 3. For each *P*, HIV load is normalized to its value at *η* = 0.9 (white dashed line). Here *R*_0_^pop^ = 6.25; all other parameters are as shown in Tables 1 and 2.
(PDF)

S3 Fig HIV transmission and fitness for different *η*, *P* and *κ*. Heatmaps corresponding to Fig 2B in the main text. For each *P* in A-C, values are normalized to equal 1 along the vertical white dashed line. (A) Normalized HIV transmission rate as a function of *η* for each *P*, when the composite ‘waste’ parameter *κ* is set to equal 0.01 (see the Section A S1 Text or Table 2 for a definition of *κ*). (B) Normalized HIV fitness in individual hosts as a function of *η* for each *P*, when *κ* is again set to equal 0.01. While the transmission rate is highest at *η* close to 1, fitness increases monotonically with *η* (see also Fig 2B of the main text). This monotonic dependence on *η* occurs for any *P*, although the optimal transmission may vary slightly with *P*. (C) Relative transmission rate as a function of *η* for each *P*, with *κ* now set to equal 0. Note the reduced stability region as the peak shifts up and to the right. In these figures, *R*_0_^pop^ = 6.25; all other parameters are as shown in Tables 1 and 2.
(PDF)

S4 Fig Initial success of TIP-resistant HIV mutants depends on the strength of the transcriptional asymmetry (*P*) and the presence of host-scale selection. Prevalence of HIV and TIP sub-populations following the introduction of a TIP-resistant HIV mutant with *η*_mut_ = 1 into a population co-infected with TIP and a wild-type HIV strain with *η*_wt_ = 2. Four cases are shown: (A) *P* < *P*_c_ (i.e., *P = 2*.*5*) and the *absence* of HIV multi-strain co-infections in individual hosts, (B) *P* < *P*_c_ but the *presence* of HIV multi-strain co-infection, (C) *P* > *P*_c_ (i.e., *P = 6*) but the *absence* of HIV multi-strain co-infection, and (D) *P* > *P*_c_ and the *presence* of HIV multi-strain co-infection. When *P* < *P*_c_ (i.e., A, B), TIP-resistant mutants can make TIP interventions unstable; further, when co-infection is absent (i.e., A, C) TIP-resistant mutants can persist in the population. However, the combination of *P > P*_c_ and within-host co-infection (i.e., Panel D) results in the stability of TIPs against HIV mutation, and the extinction of TIP-resistant HIV mutants (due to within-host competitive exclusion by more-fit, higher *η* strains). See Tables 1 and 2 for a list of the fixed parameters and their values.
(PDF)

S5 Fig In an HIV population with *η*_wt_ < *η*_*c*_, mutants with *η*_*mut*_ closer to *η*_*c*_ are selected for. The same simulations as in the panels of S4 Fig, except that the wild type HIV strain now has a smaller capsid/genome ratio: *η*_wt_ = 0.5 instead of 1.5. The mutant strain still has *η*_mut_ = 1. Unlike in S4 Fig, there is no evolutionary conflict between the population scale and the host scale. Selection on both scales pushes HIV towards higher values of *η*. The presence of co-infection does not alter the direction of evolution, but only expedites the outcome. Still, *P* > *P*_*c*_ = 3 prevents TIP extinction when the HIV mutant invades, and in Panel D the final prevalence of singly-HIV infected individuals becomes less than before the mutant’s introduction. Parameters: *R*_0_^pop^ = 6.25, *η*_wt_ = 0.5, *η*_mut_ = 1, *P* = 2.5 in (A,B) and *P* = 6 in (C,D). Tables 1 and 2 contain the remaining parameter values.
(PDF)

S6 Fig Long-term oscillations in the prevalence of HIV mutants result from frequency-dependent (group-selection) effects. The relative fitness of the two HIV strains is dependent upon the prevalence of TIP infected hosts, which is in turn dependent on the relative frequencies of the two HIV strains. As a result, we see oscillations as the mutant takes over (see Discussion). Parameters: *R*_0_^pop^ = 6.25, *P* = 2.5, *η*_wt_ = 2, *η*_mut_ = 1; all other parameters are contained in Tables 1 and 2.
(PDF)

S7 Fig Changes in the homeostatic shutdown function have only weak effects on the steady-state cell division rate (*h*_eq_). (A,C) The dependence of the maximum cell division rate (*h*_0_), relative target cell level (*T)*, and mean TIP copy number per cell (*q*) on the equilibrium division rate *h*_eq_ (see Eqs. 51–58 in S1 Text). (B,D) Two forms of the homeostatic shutdown function. The dependence of *h*_eq_ on *h*_0_ is dominated by the asymptotic behavior of *T* as *q* approaches 1 (i.e., as the TIP copy number increases), and is robust to the shape of homeostatic shutdown function *f*(*x*). Parameters: *η* = 0.5, *P* = 12, *R*_00_ = 10; all other parameters are found in Table 2.
(PDF)

S8 Fig TIP dynamic and evolutionary stability do not hinge on the suppression of HIV viral loads or transmission. (A) TIP stability when HIV viral loads and transmission are assumed to suppressed by TIPs. (Left panel) HIV+TIP- prevalence, (middle panel) HIV+TIP+ prevalence, and (right panel) invasion analysis of HIV mutants performed as in Fig 3C. (B) TIP stability when HIV viral loads and transmission are assumed to be unsuppressed by TIPs. (Left panel) HIV+TIP- prevalence, (middle panel) HIV+TIP+ prevalence, and (right panel) invasion analysis of HIV mutants performed as in Fig 3C. Both the prevalence of HIV+, TIP- hosts and the evolutionary stability of TIPs remain virtually unchanged despite the absence of HIV suppression, because the additional transmission of HIV is matched by the increased spread of the TIP.
(PDF)

S9 Fig Transmission rates, rather than death rates, are the key determinants of HIV transmission potential. (A) Transmission potential assuming constant host death rates. (B) Transmission potential with changing death rates.
(PDF)
