# Supplementary material for: Conflicting Selection Pressures Will Constrain Viral Escape from Interfering Particles: Principles for Designing Resistance-Proof Antivirals
Source: PLoS Comput Biol. 2016 May 6;12(5):e1004799. doi: 10.1371/journal.pcbi.1004799 (PMC4859541; doi:10.1371/journal.pcbi.1004799)
Supplement: S1 Text — Derivations and non-dimensionalizations of the cell-scale, patient-scale and population-scale mathematical models. TIP invasion and dynamic stability conditions are derived at each scale, as is the condition for the evolutionary stability of the TIP to (unilateral) HIV mutations in η. Further, sensitivity analyses are performed to test model robustness. (PDF) [file pcbi.1004799.s001.pdf]

Supplemental Information for
Conflicting Selection Pressures Will Constrain Unilateral Viral Escape from Interfering Particles: Principles for Designing Resistance-Proof Antivirals

Luke I. Rast, Igor M. Rouzine, Ganna Rozhnova, Lisa Bishop, Ariel D. Weinberger, and Leor S. Weinberger

Contents

A HIV and TIP in a single cell: the capsid-stealing model 2
Basic equations and biological interpretation 2
Output: total amounts of HIV and TIP particles produced by an infected cell 2

B HIV and TIP in individual patients 3
Basic equations and biological interpretation 3
Output: HIV and TIP loads in a host 4
Basic equations in dimensionless units 5
Dynamic stability of TIP 6
Homeostatic regulation of division 7
Effect of the homeostatic shutdown of division on TIP-free steady state 7
Effect of the homeostatic shutdown of division on TIP+ steady state 8

C TIP and HIV in a population 9
Basic equations and epidemiological interpretation 9
Link to the individual-host and single-cell scales 10
Steady-state prevalence of singly and dually infected individuals 10
Dynamic equations in dimensionless form 11
Condition for TIP spread and stability in a population 12

D Stability of TIP treatment under HIV Evolution 13
Direction of evolution and the fitness 13
Direction of evolution in a host 13
Direction of evolution in a population 14
Evolution from $\eta < \eta_c$ towards η_c 15

E Robustness Tests 16
TIP pre-infects individuals 16
Sensitivity to κ 17
Timing of HIV Transmission 17

F Supplemental Discussion 18
Rate of Super-Infection 18
Single Resource Stealing 18
Uniformity of Wild-Type Population 18
TIP toxicity 18

In Sections A-C, we describe the model of TIP and HIV dynamics at 3 linked scales of biological organization using the approach introduced in [5]. The single-cell model is adopted from [9], and is described briefly (Section A). The host-level model is slightly modified from [9], described in Section B. Population scale parameters and model are discussed in Section C, following an SIR framework modified from [5]. Section D addresses the issue of evolutionary stability of HIV under treatment, using a method from [9] for host scale, and similar eigenvalue comparisons for the population scale. Finally, various assumptions and sensitivity tests that were made are discussed in Sections E and F.
Notation: in this supplement, we use () for grouping, and [] for arguments to a function.

A HIV and TIP in a single cell: the capsid-stealing model

Basic equations and biological interpretation

At the scale of a single cell, we adopt the previously described capsid-stealing model [9] (Fig 1A). We model a cell with an integrated HIV provirus. Of all virus products, we focus on two: $C(t)$, the amount of fully formed capsids that do not yet carry genomic mRNA dimers, and $G(t)$, the amount of dimers of genomic mRNA. The system of equations has the form

$$\frac{dG}{dt} = \theta - k_{\mathrm{pck}}GC - \alpha G \tag{1}$$
$$\frac{dC}{dt} = \eta\theta - k_{\mathrm{pck}}GC - \beta C \tag{2}$$

Initial model parameters are: θ, the linear production rate of HIV genomes; k_{pck}, packaging efficiency; α and β, the exponential rates of genome and capsid loss, respectively; and η, the capsid-to-genome production ratio.

In reality, maturation of virions is a gradual process (see for review [4]) involving about 2500 Gag-Pol precursor molecules, of which half form the capsid, and half are left as a filler. In general, such processes are described by a more complex system of age-structured equations describing concentrations of RNA genomes, free proteins, and immature capsids. For the aim of our study, we consider a steady state process for which the existence of time delays between these stages of capsid formation is not essential. Concentrations of Gag-Pol precursors and capsids are proportional to each other. The term $k_{\mathrm{pck}}GC$ in Eqs. 1-2 describes the limiting step of capsid formation, the initial Gag-Pol to HIV RNA binding. The unit of "one capsid" (production rate $\eta\theta$) corresponds to the equivalent amount of 2500 Gag-Pol. Thus, the model applies after a unit adjustment.

In a cell infected with HIV provirus and co-infected with m copies of a TIP provirus, TIP genomic mRNA is also produced, and has concentration $G_{\mathrm{TIP}}(t)$. The system of equations then takes the form:

$$\frac{dC}{dt} = \eta\theta - k_{\mathrm{pck}}(G_{\mathrm{HIV}} + G_{\mathrm{TIP}})C - \beta C \tag{3}$$
$$\frac{dG_{\mathrm{HIV}}}{dt} = \theta - k_{\mathrm{pck}}G_{\mathrm{HIV}}C - \alpha G_{\mathrm{HIV}} \tag{4}$$
$$\frac{dG_{\mathrm{TIP}}}{dt} = mP\theta - k_{\mathrm{pck}}G_{\mathrm{TIP}}C - \alpha G_{\mathrm{TIP}} \tag{5}$$

Two new model parameters are: P, the ratio of TIP to HIV genome production rates, and multiplicity of TIP infection, m, an integer number, $m = 1, 2, 3, \ldots$. Eqs 1-2 can be obtained from Eqs 4-5 as the particular case with $P = 0$.

Output: total amounts of HIV and TIP particles produced by an infected cell

In order to connect to HIV and TIP dynamics at the level of an individual patient, we need to predict the burst size (total number of particles produced per cell lifetime) of HIV in singly and dually infected cells,

and that of TIP in dually infected cells. Based on previous analysis [5], we assume that steady-state viral production is reached shortly after the cell is infected and long before the death of the infected cell. Then, the total numbers of virus particles per cell are given by

$$n = (k_{\text{pck}}/\delta) G_{\text{HIV}} C|_{P=0} \tag{6}$$

$$\psi_m n = (k_{\text{pck}}/\delta) G_{\text{HIV}} C, \quad m \geq 1 \tag{7}$$

$$\rho_m \psi_m n = (k_{\text{pck}}/\delta) G_{\text{TIP}} C, \quad m \geq 1 \tag{8}$$

Here n is the HIV burst size from a cell infected with HIV only (the case obtained by setting $P = 0$), ψ_m shows decrease in HIV burst size due to co-infection with TIP, and ρ_m is the ratio of TIP to HIV burst size in a co-infected cell. The parameter $1/\delta$ is the average lifetime of an HIV-infected cell.

Solving through (see also [9], SI), the three burst size outputs (n, ψ_m, ρ_m) are given by:

$$n = \frac{\theta}{\delta} \left. \frac{y}{1+y} \right|_{P=0} \tag{9}$$

$$\psi_m n = \frac{\theta}{\delta} \frac{y}{1+y}, \quad m \geq 1 \tag{10}$$

$$\rho_m = mP, \quad m \geq 1 \tag{11}$$

$$y \equiv \frac{1}{2\kappa} \left(-(mP + 1 - \eta + \kappa) + \sqrt{(mP + 1 - \eta + \kappa)^2 + 4\eta\kappa} \right) \tag{12}$$

$$\kappa \equiv \frac{\alpha\beta}{\theta k_{\text{pck}}} \tag{13}$$

Here y is the rescaled capsid number and κ is the composite "waste parameter" characterizing the loss of HIV genomes and capsids before they form virions. If κ is small, it is equal to the lost fraction of either genomes or capsids, whichever product is limiting (i.e., loss of genomes for $\eta > 1$ and of capsids for $\eta < 1$). In this limit, the expressions above become:

$$n = \begin{cases} \frac{\theta}{\delta}, & \eta > 1 \\ \eta \frac{\theta}{\delta}, & \eta < 1 \end{cases} \tag{14}$$

$$n\psi_m = \begin{cases} \frac{\theta}{\delta}, & \eta > 1 + mP \\ \frac{\theta}{\delta} \frac{\eta}{1+mP}, & \eta < 1 + mP \end{cases} \tag{15}$$

$$\rho_m = mP \tag{16}$$

The output quantities passed to the next, higher scale of modeling are the three burst sizes in Eqs 6-8. Input parameters that affect these burst sizes are mP, η, and κ. (Parameter θ/δ , which determines the absolute burst sizes, is absorbed in the host-scale reproduction ratio; TIP copy number m is a running index at the host scale, see the next section.) Throughout the text, we take small $\kappa = 0.01$, based on the analysis in [9]. For the sensitivity of the results to κ see Section E below. Thus, there are only two variable input parameters on the scale of a single cell, P and η.

B HIV and TIP in individual patients

Basic equations and biological interpretation

We begin with the standard model of HIV-host dynamics *in vivo* [8] generalized to include production of TIP particles. The generalized model [9] includes co-infection of cells with TIP and HIV, so that dually infected cells produce less HIV. As compared to the previous version in [9], we also include division of uninfected

cells and cells infected with TIP only. Based on results of *in vivo* studies [1] (super-infection protection), we allow only a single HIV provirus per cell but multiple copies of the TIP provirus.

The system of equations has the form:

$$\frac{dT_0}{dt} = b - (d(1-h) + kV_H + kV_T)T_0 \tag{17}$$

$$\frac{dT_m}{dt} = kV_T T_{m-1} - (d(1-h) + kV_H + kV_T)T_m, \quad m \geq 1 \tag{18}$$

$$\frac{dI_m}{dt} = kVT_m - \delta I_m, \quad m \geq 0 \tag{19}$$

$$\frac{dV_H}{dt} = n\delta I_0 + n\delta \sum_{m=1}^{\infty} \psi_m I_m - cV_H \tag{20}$$

$$\frac{dV_T}{dt} = n\delta \sum_{m=1}^{\infty} \rho_m \psi_m I_m - cV_T \tag{21}$$

Here, the state variables are: T_0, uninfected CD4 T cells permissive for viral replication; $T_m, m \geq 1$, CD4 T cells harboring m copies of TIP provirus but not infected with HIV; I_0, cells infected with HIV only; $I_m, m > 1$, dually infected cells harboring a copy of HIV and m copies of TIP provirus; V_H, HIV load (free virus concentration in peripheral blood plasma); V_T, TIP load.

The model parameters, described in the literature (see [9] and refs therein) are: b, linear production rate of uninfected cells; d, natural death rate of uninfected cells; h, average number of cell divisions in the lifespan of an uninfected cell; k, infectivity factor; and δ, death rate of singly and dually infected cells. There are three additional parameters in the presence of TIP, derived at the single-cell level (Section A): n, HIV burst size from a singly infected cell; $n\psi_m$, HIV burst size from a dually infected cell with m copies of TIP provirus; and $n\psi_m\rho_m$, TIP burst size from a dually infected cell with m copies of TIP provirus.

In general, as we describe below, the number of cell divisions (h) does not have to be constant, and depends on state variables, such as the total number of cells, including uninfected and TIP-infected cells. For the next three subsections, we model h as constant. We then introduce its dynamics as corrections to the model.

The biological interpretation of Eqs 17-21 is as follows. Uninfected cells that are permissive for viral replication (T_0) are replenished from a constant source and by division. Permissive cells are depleted by three competing processes: (i) their natural death, (ii) infection by HIV particles, (iii) infection by TIPs (Eq 17). Cells that become infected by HIV (I_0) produce viral particles and die at average rate $\delta \sim 1/$ day (Eq 19, $m = 0$). Alternatively, before becoming infected with HIV, a cell can be infected with one or more copies of TIP provirus and we classify these cells according to the copy number of TIP proviruses by cell bins T_m (Eq 18). Cells infected with TIP alone do not express HIV proteins and die at the same rate as uninfected cells. If a TIP cell is subsequently infected with HIV, the cell becomes dually infected ($I_m, m \geq 1$) and begins producing both HIV and TIP particles (Eqs 20, 21). These dually infected cells are HIV+TIP+ and die as rapidly as infected cells which are HIV+TIP-, I_0. HIV particles are generated from both singly and dually HIV-infected cells (Eq 20), while TIP particles are generated from dually infected cells only (Eq 21).

Output: HIV and TIP loads in a host

Chronic HIV infection represents an approximate steady state [8]. In agreement with this fact, Eqs 17-21 have a stable steady-state solution. Setting their right-hand sides equal to zero and solving gives the steady-state:

$$\bar{T}_0 = \frac{b}{d(1 - h + \hat{V}_H + \hat{V}_T)} \tag{22}$$

$$\bar{I}_0 = \frac{d\hat{V}_H \bar{T}_0}{\delta} \tag{23}$$

$$\bar{T}_m = \bar{T}_0 q^m, \ \ m \geq 1 \tag{24}$$

$$\bar{I}_m = \bar{I}_0 q^m, \ \ m \geq 1 \tag{25}$$

$$1 + \hat{V}_H + \hat{V}_T - h = R_0^{\text{host}} \left(1 + \sum_{m=1}^{\infty} \psi_m q^m\right) \text{ or } \hat{V}_H = 0 \tag{26}$$

$$(1 + \hat{V}_H + \hat{V}_T - h)^2 = \hat{V}_H R_0^{\text{host}} \sum_{m=1}^{\infty} \rho_m \psi_m q^{m-1} \text{ or } \hat{V}_T = 0 \tag{27}$$

where, for tractability, the following new notation is used

$$R_0^{\text{host}} \equiv \frac{nkb}{cd} \tag{28}$$

$$\hat{V}_H \equiv \frac{k\bar{V}_H}{d}, \ \hat{V}_T \equiv \frac{k\bar{V}_T}{d} \tag{29}$$

$$q \equiv \frac{\hat{V}_T}{1 - h + \hat{V}_H + \hat{V}_T} \tag{30}$$

Here R_0^{host} is the basic reproduction ratio at the beginning of infection, $\hat{V}_H$ and $\hat{V}_T$ are rescaled HIV and TIP loads, and $1/(1-q)$ is the average number of integrated TIP provirus copies $\langle m \rangle$ in a dually infected cell. In the absence of TIP ($\hat{V}_T = 0$), the rescaled HIV virus load is $\hat{V}_H = R_0^{\text{host}} - 1$.

The quantities that carry forward to the population scale (Section C) are the rescaled HIV and TIP loads, $\hat{V}_H$ and $\hat{V}_T$. Calculated from Eqs 26, 27, and 30, they depend on four parameters, $R_0^{\text{host}}, P, \eta$, and κ. S1 Fig shows TIP and HIV loads rescaled to the TIP-free HIV load, as functions of (P, η) for $R_0^{\text{host}} = 10$ [7] and $\kappa << 1$.

Basic equations in dimensionless units

For the next sections, for ease of calculation and comparison between various values of η, we make the state variables unitless and introduce some new notation.
Making the substitutions:

$$t \to \frac{t}{\delta} \tag{31}$$

$$V_H \to \frac{d}{k} V_H \tag{32}$$

$$V_T \to \frac{d}{k} V_T \tag{33}$$

$$T_m \to \frac{c\delta}{k\theta} T_m \quad m = 0, 1, \ldots \tag{34}$$

$$I_m \to \frac{c\delta}{k\theta} I_m \quad m = 0, 1, \ldots \tag{35}$$

makes all of the state variables unitless.

Next, we redefine the host level variables in order to remove dependence of the host-level parameter, R_0, on the cell-level parameter, η. The new notation is:

$$\phi_m = \frac{\delta}{\theta} n\psi_m = \frac{y}{1+y}\bigg|_m \tag{36}$$

$$R_{00} = \frac{\theta}{n\delta} R_0^{\text{host}} = R_0^{\text{host}}/\min[1,\eta] = \frac{\theta \text{kb}}{\text{cd}\delta} \tag{37}$$

This removes the η dependence from R_0. Only the burst size terms, which have the form $\frac{\theta}{\delta}\phi_m$, depend on η. This makes comparison between various values of η easier, but does change the intuitive meaning of R_0 to a normalized value of the influx of T cells, rather than the (more meaningful) reproduction ratio for HIV infection of cells.
The new dimensionless system is:

$$\frac{dT_0}{dt} = \frac{d}{\delta}(R_{00} - (1 - h + V_H + V_T)T_0) \tag{38}$$

$$\frac{dT_m}{dt} = \frac{d}{\delta}(V_T T_{m-1} - (1 - h + V_H + V_T)T_m), \quad m \geq 1 \tag{39}$$

$$\frac{dI_m}{dt} = \frac{d}{\delta} T_m V_H - I_m, \quad m \geq 0 \tag{40}$$

$$\frac{dV_H}{dt} = \frac{c}{\delta}\Big(\frac{\delta}{d}\sum_{m=0}^{\infty} \phi_m I_m - V_H\Big) \tag{41}$$

$$\frac{dV_T}{dt} = \frac{c}{\delta}\Big(\frac{\delta}{d}\sum_{m=1}^{\infty} \rho_m \phi_m I_m - V_T\Big) \tag{42}$$

and has four parameters: two expressing the separation between various timescales ($\frac{c}{\delta}$ and $\frac{d}{\delta}$), and two expressing the strengths of the inputs of T-cells from various sources (R_{00} and h).

Dynamic stability of TIP

If the TIPs are to invade a population of HIV infected cells, then when a small number of TIPs are introduced into a TIP-free steady state, that number should grow, turning the steady state into an unstable equilibrium point. If we set $V_T = I_m = T_m = 0$ for m > 0 (TIP free), the steady state equations become (with limits as κ becomes small on the right)

$$T_0 = \frac{1}{\phi_0} = \begin{cases} 1, & \eta > 1 \\ 1/\eta, & \eta < 1 \end{cases} \tag{43}$$

$$V_H = R_{00}\phi_0 - 1 + h = \begin{cases} R_{00} - 1 + h, & \eta > 1 \\ \eta R_{00} - 1 + h, & \eta < 1 \end{cases} \tag{44}$$

$$I_0 = \frac{d}{\delta}\frac{1}{\phi_0}(R_{00}\phi_0 - 1 + h) = \begin{cases} \frac{d}{\delta}\left(R_{00} - 1 + h\right), & \eta > 1 \\ \frac{d}{\delta}\left(R_{00} + \frac{h-1}{\eta}\right), & \eta < 1 \end{cases} \tag{45}$$

Standard eigenvalue analysis shows this state to be unstable (i.e. the introduced TIP will expand) if and only if

$$\rho_1\phi_1 > \frac{1 - h + V_H}{V_H T_0} \tag{46}$$

Equivalently, for small κ:

$$\eta < 1: \qquad P > \tfrac{R_{00}\eta}{h-1} \qquad \text{and} \quad h > 1 \tag{47}$$

$$1 < \eta < 1 + P: \quad P > \tfrac{R_{00}}{\eta(R_{00}+h-1)-R_{00}} \quad \text{and} \quad \eta > \frac{R_{00}}{R_{00}+h-1} \tag{48}$$

$$\eta > 1 + P: \qquad P > \tfrac{R_{00}}{R_{00}+h-1} \tag{49}$$

This result means that we will have growth for all η if:

$$h > 1 + \frac{R_{00}}{P} \tag{50}$$

If cell division were absent ($h = 0$), these TIP stability conditions would only be satisfiable at $\eta > 1$ [9]. In animals chronically infected with SIV, a large range of variation in replenishment rates is observed from cell division as opposed to a linear source [6], as we model here. The average values for high and low infectivity classes were that one fourth to one half of cell death (including viral effects and the natural lifespan) is compensated by the division of preexisting cells. This corresponds to $h = 0.33R_{00}$ to $h = R_{00}$. Due to the division of uninfected cells and cells with integrated TIP provirus, as long as TIP transcriptional advantage is moderately high ($P > 3$), TIP is predicted to be stable in an individual host at almost any value of η (Fig 1B). First, however, we need to address the dependence of division on the number of T cells.

Homeostatic regulation of division

We showed in the previous section that introduction of T-cell division into the model is not only realistic, but also helps to reduce the sensitivity of the model to the parameter η. On the other hand, examination of Eq. 38 reveals that $h \geq 1$, if held constant, will cause T_0 to diverge when in an uninfected host. Depending upon how large the value is, this non-biological behavior can force the whole model into producing strange results even when HIV is present (for example, divergence in the average number of TIPs infecting a cell). Clearly, h cannot be assumed to be constant as the numbers of cells vary. Here, we assume that cell division shuts off homeostatically and so h decreases to zero as the number of division-competent cells (i.e. cells not infected by HIV) increases. We assume a specific form of the homeostatic shut-down function, but show that the results are relatively insensitive to this form:

$$h = h_0 f[\frac{T}{R_{00}}] \tag{51}$$

where

$$f[x] = 1 - \frac{1}{1+e^{-(x-1)/a}}, \quad a = .1 \tag{52}$$
$$T = \sum_m T_m$$
$$h_0 = 3.33 = 0.33R_{00}$$

and $x = 1$ corresponds approximately to the pre-infection level of T. Because we estimated h in equilibrium to be somewhere in the range 3-10, and $h < h_0$, this choice of h_0 is a conservative estimate of the maximal division rate.

Effect of the homeostatic shutdown of division on TIP-free steady state

Above, we calculated the steady state in terms of h assuming a constant value. The next question is what effect adjusting the value of h dynamically has on steady state levels.

For the TIP-free steady-state, the answer is simple. Because the steady state value of T_0 is independent of the value of h, it will not change upon introduction of any shutdown function. Hence, we may set

$$h = h_0 f[\frac{1}{\phi_0 R_{00}}]$$

in the derived equations (43 - 45) to give correct expressions.

Now that we have a dynamic h, the second condition in Eqn. 47 begins to take on meaning, especially if we assume (as is reasonable for a homeostatic shutdown function) that f is non-increasing.
Requiring

$$h_0 f[\frac{1}{\phi_0 R_{00}}] > 1$$

shows that, although TIPs eventually become unstable regardless of the value of h_0, higher h_0 values lead to an expanded stability region for TIP. On the other hand, if η becomes lower than a zero of $R_{00}\phi_0 + h - 1$, then HIV will become unstable. As a result, as we increase the value of h_0, the region of both HIV stability and TIP instability becomes smaller. For example, for the shutdown function in eqn. 52 and $h_0 = 3.33$, the region is: $\eta \in [.079, .092]$, as compared to $\eta \in [.1, 1]$ for $h_0 = 0$.

Effect of the homeostatic shutdown of division on TIP+ steady state

The steady-state values with TIP are harder to solve for. We must truncate the infinite sequence of T_m and I_m values at some m. This will result in m possible stead-state solutions, but very few of them will correspond to biologically relevant scenarios. For example, in equation 30, nonzero values of h open the door for negative values of q, which are non-biological because they correspond to negative T_i when i is even. The large number of branches and small number of relevant ones means that generic solvers will often choose the wrong branch, and so produce non-biological results. To deal with such complications, we used a custom numerical scheme:
We start from

$$T_0 = \frac{R_{00}}{1 - h + V_H + V_T}$$

$$I_0 = \frac{d}{\delta} V_H T_0$$

$$T_m = T_0 q^m, \ m \geq 1$$

$$I_m = I_0 q^m, \ m \geq 1$$

$$1 - h + V_H + V_T = R_{00} \sum_{m=0}^{\infty} \phi_m q^m \text{ or } V_H = 0$$

$$(1 - h + V_H + V_T)^2 = V_H R_{00} \sum_{m=1}^{\infty} \rho_m \phi_m q^{m-1} \text{ or } V_T = 0$$

$$q = \frac{V_T}{1 - h + V_H + V_T}$$

First, we solve for q. Making the biological assumptions that the V_H, V_T, and $\sum_m T_m$ are finite means that $q \in [0, 1)$, which allows us to truncate the sums at a sufficiently large index, producing polynomials in q.
Using the notation:

$$P_1[q] = R_{00} \sum_{m=0}^{m_{\max}} \phi_m q^m \tag{53}$$

$$P_2[q] = R_{00} \sum_{m=1}^{m_{\max}} \rho_m \phi_m q^{m-1} \tag{54}$$

$$\alpha = 1 - h + V_H + V_T \tag{55}$$

produces the equations (noting that for $q \in [0,1)$, $P_2 > 0$):

$$\alpha = P_1[q] \tag{56}$$

$$\alpha^2 = V_H P_2[q] \Rightarrow V_H = \frac{P_1[q]^2}{P_2[q]} \tag{57}$$

So,

$$0 = P_2[q]((1-q)P_1[q] - 1 + h) - P_1[q]^2 \tag{58}$$

This polynomial is then solved numerically, and the solutions can be filtered to put constraints on q (namely, q is real and $q \in [0,1)$). Because each value of q corresponds to a branch of the system, this effectively allows us to focus only on the relevant branches. Once q has been solved for, Eqs. 55, 56 and 57 allow us to solve for V_H and V_T, and from there, for all T_i and I_i values.

In order to introduce the homeostatic shutdown function, we back-calculate from a value of h at steady-state to determine the system that it corresponds to. We:

1. Assume a steady-state value of $h = h_{\text{eq}}$ and use this to solve the polynomial, producing the value of q at steady-state.

2. Given q, we calculate the steady-state T-cell count:

$$T = \sum_m T_m = \frac{R_{00}}{P_1[q](1-q)}$$

3. Finally, using our specific shutdown function, we back-calculate h_0 as:

$$h_0 = \frac{h_{\text{eq}}}{f[T/R_{00}]}$$

The plot of q, T, and h_0 against the equilibrium division rate h_{eq} is shown in S7 Fig. These plots help to highlight why there is only a weak dependence of the results on the rolloff function: because its only influence is to slightly adjust the h_0 vs h_{eq} dependence around the T solution.

This method gives results that we can be confident in, but makes surveying large areas of parameter space for given h_0 somewhat cumbersome. We used a variable step size method to find bounds on the required values of h_{eq}, after which desired accuracy was obtained with a bisection method.

C TIP and HIV in a population

Basic equations and epidemiological interpretation

We consider a uniform unstructured population with virus transmission between patients during the chronic phase of HIV infection. According to a standard SIR approach, it is represented by the system of equations

$$\frac{dS}{dt} = \lambda - \frac{c}{N}\beta_H^I\ S\ I - \frac{c}{N}\beta_H^{I_D}\ S\ I_D - \delta_S\ S \tag{59}$$

$$\frac{dI}{dt} = \frac{c}{N}\beta_H^I\ S\ I + \frac{c}{N}\beta_H^{I_D}\ S\ I_D - \frac{c}{N}\beta_T^{I_D}\ I\ I_D - \delta_I\ I \tag{60}$$

$$\frac{dI_D}{dt} = \frac{c}{N}\beta_T^{I_D}\ I\ I_D - \delta_D\ I_D \tag{61}$$

The epidemiological processes included are as follows: Susceptible individuals, whose number is denoted S, enter the high-risk group at linear rate λ and leave it with exponential rate δ_S. They can be infected

with HIV and become singly infected. The singly infected individuals, I, can be superinfected with TIP and become dually infected individuals, I_D. Both singly infected and dually infected individuals can transmit HIV further, although at different rates (see below). Only dually infected individuals can transmit TIP. The model in Eqs 59-61 represents a simplified version of the one studied in [5].

The model parameters are the frequency of contacts per unit time c, the death rates $\delta_S, \delta_I, \delta_D$ of the three groups of individuals, and the three transmission (dimensionless) coefficients $\beta_H^I, \beta_H^{I_D}, \beta_T^{I_D}$. In what follows, we take the frequency of contacts, c, to be linearly increasing with the total population, N. This corresponds to an assumption of density dependent interactions within the population. Given this assumption, the quantity c/N is a constant.

Link to the individual-host and single-cell scales

These parameters can be expressed in terms of HIV and TIP virus loads based on epidemiological data [2,5]:

$$\beta_H^I = F[V_H[\eta, P=0]] \tag{62}$$
$$\beta_H^{I_D} = F[V_H[\eta, P]] \tag{63}$$
$$\beta_T^{I_D} = F[V_T[\eta, P]] \tag{64}$$
$$F[V] \equiv \frac{0.54V}{4.14+V} \tag{65}$$
$$\delta_I = D[V_H[\eta, P=0]] \tag{66}$$
$$\delta_D = D[V_H[\eta, P]] \tag{67}$$
$$\delta_S = \frac{1}{35} \tag{68}$$
$$D[V] = \left(\frac{25.4 \times (0.35)^{0.41}}{(0.35)^{0.41} + V^{0.41}}\right)^{-1} \tag{69}$$

where $V_H[\eta, P]$ and $V_T[\eta, P]$ are the HIV and TIP loads, respectively, in units of 10^5 RNA copies/ml blood. In Eq 62, the case $P = 0$ corresponds to the absence of TIP. Virus loads are expressed in terms of single-cell parameters η and P (Section B; S1 Fig). The average HIV viral load in the absence of TIP is $\bar{V_H}(P=0) = 10^5$ RNA copies/ml, for which we get $\beta_H^{I_D} = \beta_1 = 0.105$.

Steady-state prevalence of singly and dually infected individuals

In the absence of HIV infection, from Eq 59, the number of uninfected individuals remains at the steady-state level

$$S^0 = \frac{\lambda}{\delta_S}, \tag{70}$$
$$I = I_D = 0 \tag{71}$$

In what follows, it will be convenient to rescale the 3 state variables to units of S^0:

$$\hat{S} = \frac{S}{S^0},\ \hat{I} = \frac{I}{S^0},\ \hat{I}_D = \frac{I_D}{S^0} \tag{72}$$

In the presence of HIV infection, but before introduction of TIP, the rescaled steady-state subpopulations are

$$\hat{I} = \frac{1}{B}(1 - 1/R_0^{\text{pop}}) \tag{73}$$
$$\hat{S} = 1/R_0^{\text{pop}} \tag{74}$$
$$\hat{I}_D = 0 \tag{75}$$

where

$$R_0^{\text{pop}} \equiv \frac{\lambda c \beta_1}{N \delta_I \delta_S} \tag{76}$$

$$B \equiv \frac{\delta_I}{\delta_S} \tag{77}$$

The prevalence of infection $x = I/(I + S + D)$ is related to the reproduction ratio:

$$R_0^{\text{pop}} = 1 + \frac{\delta_I x}{\delta_S (1 - x)} \tag{78}$$

Considering the presence of both HIV and TIP, we obtain

$$\hat{S}^{ss} = \frac{1}{B\tau/\phi + \mu R_0^{\text{pop}} B \hat{I}_D + 1} \tag{79}$$

$$\hat{I}^{ss} = \frac{\tau}{R_0^{\text{pop}} \phi} \tag{80}$$

$$(\hat{I}_D^{ss})^2 + \hat{I}_D^{ss} \frac{1}{R_0^{\text{pop}} B} \left(\frac{B\tau}{\phi\mu} + \frac{B}{\phi} + \frac{1}{\mu} - \frac{R_0^{\text{pop}}}{\tau} \right) + \frac{1}{(R_0^{\text{pop}})^2 \phi^2 \mu B} \Big(B\tau - \phi(R_0^{\text{pop}} - 1) \Big) = 0 \tag{81}$$

where we use R_0^{pop} and B as in Eqs 76 and 77 and define the three new parameters:

$$\mu = \frac{\beta_H^{I_D}}{\beta_1}, \ \mu < 1 \tag{82}$$

$$\phi = \frac{\beta_T^{I_D}}{\beta_1}, \ \phi > 1 \tag{83}$$

$$\tau = \frac{\delta_D}{\delta_I}, \tau < 1 \tag{84}$$

Note that the parameters μ and ϕ represent the relative transmission rates of HIV and TIP in dually infected individuals in units of the base HIV transmission rate, and that $1/\tau$ represents the increase that TIP confers to the lifespan of an HIV-infected individual.

To link this back to the single-cell parameters η, κ, P, we express μ, ϕ, τ in terms of HIV and TIP loads from Eqs 62-69, and then substitute the virus loads calculated in Section B as functions of η, P, κ. HIV prevalence in a steady state population as a function of η, P at fixed $\kappa = 0.01$ is shown in Fig 2A.

Dynamic equations in dimensionless form

In the new notation, the dynamic equations, Eqs 59-61, take the form

$$\frac{1}{\delta_I} \frac{d\hat{S}}{dt} = (1/B)(1 - \hat{S}) - R_0^{\text{pop}} (\hat{S} \ \hat{I} + \mu \ \hat{S} \ \hat{I}_D) \tag{85}$$

$$\frac{1}{\delta_I} \frac{d\hat{I}}{dt} = R_0^{\text{pop}} (\hat{S} \ \hat{I} + \mu \ \hat{S} \ \hat{I}_D - \phi \ \hat{I} \ \hat{I}_D) - \hat{I} \tag{86}$$

$$\frac{1}{\delta_I} \frac{d\hat{I}_D}{dt} = R_0^{\text{pop}} \phi \ \hat{I} \ \hat{I}_D - \tau \ I_D \tag{87}$$

which, in addition to the parameters affecting the steady state, depend on time scale $1/\delta_I$.

Condition for TIP spread and stability in a population

To find general conditions for the spread of TIP, we start from reexamining the steady state. The biologically relevant solution to the quadratic equation in Eq. (81) has the form

$$\hat{I}_D^{ss} = \frac{1}{2}\Big(- X + \sqrt{X^2 + 4Y}\Big) \tag{88}$$
$$X = \frac{1}{R_0^{\text{pop}} B}\left(\frac{B\tau}{\phi\mu} + \frac{B}{\phi} + \frac{1}{\mu} - \frac{R_0^{\text{pop}}}{\tau} \right)$$
$$Y = \frac{1}{(R_0^{\text{pop}})^2 \phi^2 \mu B}\big(\phi(R_0^{\text{pop}} - 1) - B\tau\big)$$

Regardless of sign of X, the dually infected fraction is positive, $\hat{I}_D > 0$, if $Y > 0$, which holds at

$$\phi > \frac{B\tau}{R_0^{\text{pop}} - 1} \tag{89}$$

This inequality, which represents a necessary condition for the stablity of TIP, can be rewritten as

$$R_0^{\text{pop}} - \frac{B\tau}{\phi} > 1 \tag{90}$$

Next, suppose we begin in a TIP-free steady state, Eqs 73-75, and introduce a small number of dually infected individuals I_D. To test whether TIP will spread in a population further, we consider $s,\ i,\ i_D \ll 1$ as small perturbations away from the TIP-free steady state:

$$\hat{S}[t] = \hat{S}^{ss}(1 + s[t]) \tag{91}$$
$$\hat{I}[t] = \hat{I}^{ss}(1 + i[t]) \tag{92}$$
$$\hat{I}_D[t] = i_D[t] \tag{93}$$

This leads to the equation

$$\frac{di_D}{dt} = \delta_S\big(\phi(R_0^{\text{pop}} - 1) - B\tau\big)\ i_D \tag{94}$$

In order to have TIP expand in a population, we need the right-hand side to be positive and again arrive at the inequality in Eq 89.

Thus, TIP spreads and is stable under the same threshold condition, Eq 89, where the left-hand side represents the "effective reproduction ratio" of TIP. Moreover, the same condition is also clearly connected to the ratio of HIV-infected population sizes before and after TIP:

$$\frac{I[\phi = 0]}{I[\phi]} = \frac{B\tau}{\phi(R_0^{\text{pop}} - 1)} < 1 \tag{95}$$

where $\phi = 0$ corresponds to the absence of TIP, Eq 73. If we denote the percent of individuals infected with HIV in a TIP-free system as

$$x \equiv \frac{\hat{I}^{ss}}{\hat{S}^{ss} + \hat{I}^{ss}} = \frac{R_0^{\text{pop}} - 1}{B + R_0^{\text{pop}} - 1}. \tag{96}$$

then the TIP stability/spread threshold, Eq 89, can be rewritten as

$$\frac{\phi}{\tau} > \frac{1 - x}{x} \tag{97}$$

We see clearly that the threshold in ϕ is dependent only on the initial HIV prevalence, x, and the lifespan increase, τ, but does not depend (explicitly) on R_0^{pop}, B or on the relative HIV transmission rate μ. We link ϕ and τ to the single-cell parameters as explained above (after Eq 84). At fixed κ, R_0^{pop} and x, TIP stability can be shown as a region in the plane (η, P) (Fig 1B, C in the main text). As we observe, at high prevalence, $x > 0.5$, the population-level threshold is quite close to the host-level threshold.

D Stability of TIP treatment under HIV Evolution

Direction of evolution and the fitness

The fitness of a virus strain is determined by the average progeny number, i.e. the average number of new infections resulting from an individual infected cell. At steady state, the average progeny number is equal to one. If a mutation occurs, the mutant strain will have a smaller or larger average progeny number; the relative difference is referred to as the selection coefficient s_{eff}. Depending on the sign of s_{eff}, the mutant will expand or contract as $\exp[s_{\text{eff}}\delta t]$ and either make a foothold in the population, or go extinct. Here, $1/\delta$ is the time interval of one generation, equal to the average lifetime of an infected cell. We assume a deterministically large population, and a single mutation with small fitness effect, as given by $|s_{\text{eff}}| \ll 1$. When the quantity of the mutant introduced is small, its introduction corresponds to a linear perturbation of the model, and so, s_{eff} corresponds to the leading eigenvalue of the Jacobian of the system [3].

For the special case in which the rate of mutant expansion or contraction is dependent only upon the difference between parameters describing the mutants (no frequency dependent effects, etc.), the system is conservative, so the fitness can be expressed as a potential function, a fitness landscape. In this case, effective selection coefficient represents the log-slope of the fitness landscape in the direction of the mutation event. In this system, a fitness landscape is sufficient to capture the behavior at the host scale, but insufficient to describe the population scale behavior. See also the main text discussion.

Note that even a beneficial mutation emerging within a genetically diverse population is likely to become extinct due to the combination of random drift and linkage effects. Indeed, a mutation must occur within a high-fitness strain to become amplified and fixed in a population. Mathematical theories have been developed to describe the fixation probabilites and the speed of evolutions [Refs 6-14 from [10]]. In the present work, we do not consider these complexities: our interest is in the general direction of evolution rather than its exact speed, and in the sign of s_{eff} as the pointer.

Direction of evolution in a host

We start from a steady-state population, with state variables given by Eqs 22-27 in Section B. Following [3], the cells, the wild-type virus, and the TIPs are in a steady dynamic equilibrium prior to introduction of the mutant, so any changes here will decay. Further, changes in these variables only enter the mutant equations as higher-order terms, so they do not effect linear perturbations of the system. Hence, the eigenvalues of the system will just be the eigenvalues of the mutant and wild-type portions of the system individually, and only eigenvalues from the mutant system have the potential to be positive (as in [3]). These correspond to the eigenvalues of:

$$\begin{bmatrix} \frac{d}{\delta}T_0^{ss} & -1 & 0 & \cdots & 0 \\ \frac{d}{\delta}T_1^{ss} & 0 & -1 & \cdots & 0 \\ \vdots & \vdots & \vdots & \ddots & \vdots \\ \frac{d}{\delta}T_m^{ss} & 0 & 0 & \cdots & -1 \\ -\frac{c}{\delta} & \frac{c}{d}\phi_0^{mut} & \frac{c}{d}\phi_1^{mut} & \cdots & \frac{c}{d}\phi_m^{mut} \end{bmatrix} \tag{98}$$

Where the top $m+1$ rows correspond to I_0^{mut} through I_m^{mut}, and the bottom row corresponds V_H^{mut}.

In this case, the direction of evolution within hosts can be encapsulated by a fitness landscape [9]. We examine a small change in η, with corresponding small fold differences in n and ψ_m (calculated from the cell scale model)

$$\begin{aligned} &n \to n(1+\Delta_n), \quad \psi_m \to \psi_m(1+\Delta_{\psi m}) \text{ i.e.} \\ &\Delta_n \equiv \partial n/n, \quad \Delta_{\psi m} \equiv \partial \psi_m/\psi_m \end{aligned} \tag{99}$$

Neglecting the second-order terms in Δ, the mutant subpopulations expand in time as $\exp[s_{\text{eff}}\delta t]$, where s_{eff} is given by

$$s_{\text{eff}} = \Delta_n + \frac{\sum_{m=1}^{\infty} \psi_m q^m \Delta_{\psi m}}{1 + \sum_{m=1}^{\infty} q^m \psi_m} \tag{100}$$

where n and ψ_m are given by Eqs 9, 10, and 12 (Section A).

Intuitively, Eq. 100 is a weighted average of fitness differences due burst sizes differences between the two mutants. The Δ_n term corresponds to the flat fitness changes due to TIP-independent changes in burst size; this applies uniformly to all infected cells. The $\Delta_{\psi m}$ term is a weighted average over all cells of TIP-associated changes to the burst size. This applies differently to cells with different numbers of TIP, so $\Delta_{\psi m}$ changes with m.

s_{eff} can also be written as the relative change in the burst size of an HIV-infected cell averaged over the TIP provirus number, m:

$$s_{\text{eff}} = \partial n_{\text{av}} / n_{\text{av}}|_{q=\text{const}} \tag{101}$$

$$n_{\text{av}} = n \frac{1 + \sum_{m=1}^{\infty} \psi_m q^m}{1 + \sum_{m=1}^{\infty} q^m} \tag{102}$$

The fitness landscape in η is shown in Fig 2B and S3B Fig. We observe that in both TIP-treated (with $P = 0$) and untreated patients, fitness increases towards larger η. The direction of selection pressure is towards making more capsids to compensate for their loss (and, in the dually infected individuals, also for their stealing by TIP). Thus, TIP-resistance mutations are selected against in a host.

Direction of evolution in a population

Plugging our model into above equations, we see that, within a single host, HIV mutants with larger value of η are uniformly selected for (Fig 2). In contrast, selection at the population level is a balance between several factors. To analyze when each of these factors is dominant, we introduce a second set of population compartments, representing infection by a different HIV strain:

$$\frac{1}{\delta_I}\frac{dS}{dt} = \frac{1}{B}(1 - S) - R_{0\,1}^{\text{pop}}(SI_1 + \mu_1 SI_{D1}) - R_{0\,2}^{\text{pop}}(SI_2 + \mu_2 SI_{D2}) \tag{103}$$

$$\frac{1}{\delta_I}\frac{dI_1}{dt} = R_{0\,1}^{\text{pop}}(SI_1 + \mu_1 SI_{D1} - \phi_1 I_1 I_{D1} + c_o(I_2 I_1 + \mu_1 I_2 I_{D1})) - R_{0\,2}^{\text{pop}}(\phi_2 I_1 I_{D2}) - \frac{\delta_{I1}}{\delta_I} I_1 \tag{104}$$

$$\frac{1}{\delta_I}\frac{dI_2}{dt} = R_{0\,2}^{\text{pop}}(SI_2 + \mu_2 SI_{D2} - \phi_2 I_2 I_{D2}) - R_{0\,1}^{\text{pop}}(\phi_1 I_2 I_{D1} + c_o(I_2 I_1 + \mu_1 I_2 I_{D1})) - \frac{\delta_{I2}}{\delta_I} I_2 \tag{105}$$

$$\frac{1}{\delta_I}\frac{dI_{D1}}{dt} = R_{0\,1}^{\text{pop}}\left[\phi_1 I_1 I_{D1} + c_o(I_{D2} I_1 + \mu_1 I_{D2} I_{D1})\right] + R_{0\,2}^{\text{pop}}(\phi_2 I_1 I_{D2}) - \tau_1 I_{D1} \tag{106}$$

$$\frac{1}{\delta_I}\frac{dI_{D2}}{dt} = R_{0\,2}^{\text{pop}}(\phi_2 I_2 I_{D2}) + R_{0\,1}^{\text{pop}}\left[\phi_1 I_2 I_{D1} - c_o(I_{D2} I_1 + \mu_1 I_{D2} I_{D1})\right] - \tau_2 I_{D2} \tag{107}$$

Here c_o gives the ratio between the probability of co-infection and the probability of naive infection. δ_{I1} and δ_{I2} are calculated as before (eqn 66). The units of time, $\delta_I = 0.1$, is taken to be a standard value across all of the equations. This standard value is used in calculating the remainder of the parameters (R_0, τ, etc), which are defined as previously. For example, $\tau_1 = \frac{\delta_D^2}{\delta_I}$ where δ_D^2 is calculated by plugging steady-state viral loads for strain 1 into Eqs. 67, and $\delta_I = 0.1$. State variables also remain the same, with the addition of different variables for each HIV strain. Strain 1 is assumed to be better-fit within a host than strain 2. When a host infected with strain 2 is superinfected with strain 1, strain 1 rapidly outcompetes strain 2. Examples of the dynamics that follow after introduction of a small mutant population are shown in Fig 3B, and S4 and S5 Figs, where $c_o = 0$ in the no co-infection case and $c_o = 1$ in the co-infection case (see Fig 3B and the discussion below).

Again we wish to ascertain the general direction of flow of η under evolution, so we use a similar analysis to the host level above: determine whether a mutant population will grow or shrink based upon the sign of the eigenvalues of the system when small amounts of mutant are introduced into the wild-type steady state. Denote the prevalences of uninfected, HIV infected, and HIV and TIP infected individuals at the wild-type steady state by S^{ss}, I^{ss}, and I_D^{ss} respectively. We take the linear components of changes resulting from perturbations in the mutant variables (the Jacobian). The wild-type is in a stable steady state prior to introduction of mutant, so any changes here will decay. Furthermore, changes in wild-type variables (and S) will only enter the mutant equations as higher-order terms. Hence, the eigenvalues of the system will just be the eigenvalues of the mutant and wild-type portions of the system individually, and only eigenvalues from the mutant system have the potential to be positive (as in [3]). For partially resistant, the mutant matrix is:

$$\delta_I \begin{bmatrix} R_{0\,2}^{\text{pop}}(S^{ss}) - R_{0\,1}^{\text{pop}}(c_o(I^{ss} + \mu_1 I_D^{ss}) + \phi_1 I_D^{ss}) - \frac{\delta_{I2}}{\delta_I} & R_{0\,2}^{\text{pop}}\mu_2 S^{ss} \\ R_{0\,1}^{\text{pop}}\phi_1 I_D^{ss} & -R_{0\,1}^{\text{pop}}c_o(I^{ss} + \mu_1 I_D^{ss}) - \tau_2 \end{bmatrix} \tag{108}$$

For fully resistant mutants, there is no I_{D2}, and the mutant matrix is (a scalar):

$$\delta_I[R_{0\,2}^{\text{pop}}S^{ss} - R_{0\,1}^{\text{pop}}c_o(I^{ss} + \mu_1 I_D^{ss}) - \frac{\delta_{I2}}{\delta_I}] \tag{109}$$

Fig 3C in the main text was calculated from maximal eigenvalues of the two matrices above (108, 109) and their analogs for mutation from I_2 to I_1.

In principle, a fitness landscape for the population can be calculated in analogy to the host-level process above (from [9]) if we are willing to neglect both frequency dependent effects and constant terms in the selection strengths (which result from co-infection, because it occurs independent of the magnitude of differences in η). Expanding in terms of the changes in parameter values resulting from a small change $\Delta\eta$ (these can be calculated from the lower levels of model) gives:

$$s_{\text{eff}} = \delta_I \frac{-\Delta_\tau(R_0 S^{ss} - R_0\phi_1 I_D^{ss} - 1) - \Delta_R(\tau_1 S^{ss} + R_0\phi_1\mu_1 I_D^{ss} S^{ss}) - \Delta_\mu(R_0^2\phi_1 I_D^{ss})S^{ss}}{R_0 S^{ss} - R_0\phi_1 I_D^{ss} - 1 - \tau_1}$$

Which could then be integrated along each fixed value of P. However, as we discussed in the main text, the major interesting evolutionary behavior of the model results from deviations from landscape behavior.

Evolution from $\eta < \eta_c$ towards η_c

In the main text, we saw that in the parameter regime $\eta < \eta_c$, the host level became evolutionarily unstable. That is to say, if the wild-type HIV is assumed to have a value of η in the range $\eta < \eta_c$ (where η_c was a critical value of η (dependent on P) at which the HIV load was maximized) then evolution would favor enhanced viral loads in individual hosts. However, we argued that this was a result of enhanced HIV efficiency rather than reduced suppression from TIP, and that selection pressures in this direction were imposed regardless of the presence of TIP. An example of this behavior is shown in S5 Fig. Note that in this case, regardless of the values of the parameters (P or scale-separation), the mutant always takes over the population. In fact, the presence of coinfection simply serves to enhance the spread of the mutant. The selection pressures at the host level and the population level are always aligned. [It is also noteworthy that, as mentioned in the main text, although the mutant maintains enhanced loads in hosts, the prevalence of individuals not exposed to TIP therapy is decreased].

This behavior is a quirk of the single-cell system used to describe capsid stealing. It is built into the model by the fact that, TIP or no TIP, HIV is always able to produce genomes at the same rate, and capsids are produced at no cost to genome production. That is to say, it is an artifact of the assumption that there is no trade-off in terms of genome production for the production of capsids. In this case, it is evidently better to always be producing more capsids if there is no competitor. It is this same effect that results in the trade-offs for reduced capsid production that we observed, and that the model was trying to capture. However, as

an artifact, when the capsid to genome production ratio gets too low, pressures towards enhancing capsid production swamp all others. A model that avoided this sort of artifact would be selectively neutral for wild-type populations of HIV prior to the introduction of TIP, and would only output selection effects that result from the change in the system. This is a limitation of the model; having a wild type which has been held at $\eta = .5$ requires either additional external selective forces or a molecular limit to evolution.

E Robustness Tests

We performed several robustness tests on the model, as follows:

TIP pre-infects individuals

The above analysis assumes that TIP infects only individuals already infected with HIV (Section C). As we mentioned in the previous subsection, TIP is expected to infect HIV-negative individuals equally well. In this subsection, we investigate an alternative model, in which the preinfection is the mode of TIP transmission. We consider the two modes separately for the sake of tractability.

Model equations for populations of susceptibles, S, TIP-only singly-infected individuals, S_T, HIV-only singly-infected individuals, I, and dually infected individuals I_D are as follows

$$\frac{dS}{dt} = \lambda - \frac{c}{N}\beta_1\ S\ I - \frac{c}{N}\beta_H^{I_D}\ S\ I_D - \frac{c}{N}\beta_T^{I_D}\ S\ I_D - \delta_S\ S \tag{110}$$

$$\frac{dS_T}{dt} = \frac{c}{N}\beta_T^{I_D}\ S\ I_D - \frac{c}{N}\beta_1\ S_T\ I - \frac{c}{N}\beta_H^{I_D}\ S_T\ I_D - \delta_S\ S_T \tag{111}$$

$$\frac{dI}{dt} = \frac{c}{N}\beta_1\ S\ I + \frac{c}{N}\beta_H^{I_D}\ S\ I_D - \delta_I\ I \tag{112}$$

$$\frac{dI_D}{dt} = \frac{c}{N}\beta_1\ S_T\ I + \frac{c}{N}\beta_H^{I_D}\ S_T\ I_D - \delta_D\ I_D \tag{113}$$

which replace Eqs 59-61 of the TIP super-infection model. The new state variable is the number of TIP-preinfected invidividuals, S_T.

As in the TIP super-infection model, Eqs 110-113, have a steady state solution. In the absence of HIV, or in the presence of HIV but in the absence of TIP, the steady states levels for S, I and I_D are the same as in the previous model [Eqs 70-71 and 73-75, respectively]. TIP-preinfected individuals are absent in either case, $S_T = 0$.

In the general case, TIP is present in a population. Using rescaling defined in Eq 72, we calculate the steady state solutions for each variable:

$$\hat{S} = \frac{\hat{I}}{R_0^{\text{pop}}(\hat{I} + \mu\hat{I}_D)} \tag{114}$$

$$\hat{S_T} = \frac{\tau\hat{I}_D}{R_0^{\text{pop}}(\hat{I} + \mu\hat{I}_D)} \tag{115}$$

$$\hat{I} = \frac{\tau}{\phi - \tau}\left(\frac{1}{R_0^{\text{pop}}B} + \mu\hat{I}_D\right) \tag{116}$$

$$\hat{I}_D^2\left(\frac{\mu}{\phi - \tau} + 1\right) + \hat{I}_D\left(-\frac{1}{B\tau} + \frac{1}{R_0^{\text{pop}}B}\left(\frac{1}{\phi - \tau} + \frac{1}{\mu} + \frac{\tau}{\phi(\phi - \tau)} + \frac{1}{\phi}\right)\right)$$
$$+\frac{1}{(R_0^{\text{pop}})^2B^2\phi\mu}\left(-R_0^{\text{pop}} + \frac{\phi}{\phi - \tau}\right) = 0 \tag{117}$$

where $R_0^{\text{pop}}, B, \mu, \phi$ and τ are defined in Eqs 76, 77, and 82-84. Eq 117 has a single positive solution if the

last term in its left-hand side is negative. The resulting condition

$$\phi > \frac{R_0^{\text{pop}} \tau}{R_0^{\text{pop}} - 1} \tag{118}$$

is the new stability condition for TIP in a population.

Suppose we begin in a TIP free system, Eqs 73-75, $S_T = 0$, and add in a small amount of TIP. We consider s, s_T, i, i_D as small perturbations away from the TIP free steady state

$$\begin{aligned} \hat{S} &= \hat{S}^{SS}(1+s) \\ \hat{S}_T &= s_T \\ \hat{I} &= \hat{I}^{SS}(1+i) \\ \hat{I}_D &= i_D \end{aligned}$$

This leads to the system

$$\frac{ds_T}{dt} = \delta_s \left(-R_0^{\text{pop}}\ s_T + \phi B\ i_D\right) \tag{119}$$

$$\frac{d\hat{i}_D}{dt} = \delta_s \left(\left(R_0^{\text{pop}} - 1\right)\ s_T - B\tau\ i_D\right) \tag{120}$$

To obtain the threshold of TIP spread, we have to determine when the determinant of the corresponding 2x2 matrix is negative. The spread threshold concides with the stability threshold in Eq 118.

We can also rewrite the stability threshold in terms of the HIV prevalence x in a TIP-free population, Eq 96:

$$\phi > \tau \left(\frac{1-x}{Bx} + 1\right) \tag{121}$$

Unlike in the TIP superinfection model where the threshold depends only on τ and x, Eq 97, now it depends also on B. As previously, it does not depend on R_0^{pop} or the relative HIV transmission rate μ. The main difference from the TIP superinfection model is that, with TIP preinfection, at moderately large values of B, the TIP stablity threshold is not very sensitive to low values of HIV prevalence (Eq 121) (Fig 1C).

Sensitivity to κ

Through-out the main text, we assumed a small, fixed value of the single-cellular waste parameter, κ following [9] who showed that regardless of the presence or absence of TIP, HIV would be pressured to evolve towards small κ. We assumed $\kappa = 0.01$, but some of the results derived were sensitive to the value of κ. In particular, the shape and height of the host instability instability region was sensitive. Decreased κ increases the height of the peak to a limit around $P = 3.5$, and moved the critical value of η slightly towards 1 (S3A and S3C Figs).

Timing of HIV Transmission

There is research [11] to suggest that much of transmission occurs during the acute phase of infection. This means that TIP-suppression of HIV transmission would decrease because HIV is transmitting before super-infection by the TIP. To test the robustness of the predictions to this model, we allowed HIV transmission from TIP+ individuals to proceed as if unsuppressed. That is to say, we set $\mu = 1$. This is a very aggressive perturbation of the model, but does not change either the prevalence of HIV+TIP- hosts in the population or the evolutionary behavior (S8 Fig). HIV+TIP+ hosts do increase in prevalence to compensate for the increased transmission of HIV.

F Supplemental Discussion

We made several modeling decisions whose relaxation could yield further interesting considerations for TIP evolution. These include:

Rate of Super-Infection

In principle, the rate of host stealing is dependent on several factors, including the probability of super-infection as compared to nieve infection, the rate of take-over of a host as compared to the waiting time between interactions with other members of the population, and chances of extinction events wiping out small populations before they can establish a foothold.

Single Resource Stealing

Capsid stealing represents a very simple interference mechanism: stealing of a single resource in trans. This could be generalized to higher number, or even n resource stealing models. Further, while [9] argued that cis-stealing of genomes was not evolutionarily stable, stealing need not be limited to either one mechanism or another. For example, the feedback between concentrations of gene products and rates of splicing could result in indirect stealing of genomes through the use of gene products.

Uniformity of Wild-Type Population

The wild-type virus was assumed to be genetically uniform. This allowed for a direct comparison of fitnesses of two HIV strains, but is quite unrealistic. Actually, HIV is quite diverse, and evolution at different sites is not independent, as it has strong linkage effects (genetic background effects and clonal interference) which attract great deal of study (see for a review [10]).

TIP toxicity

Here, we allowed arbitrarily large numbers of TIPs to co-infect individual cells prior to HIV infection. This allowed the long-lived TIP population to have any size, however large. However, we could have imposed restrictions on TIPs per cell, or feedback between the number of TIPs coinfecting and the death rates of the cells. Either of these would impose more stringent control on the TIP population, and so would make "capsid flooding" by increasing η a viable strategy for HIV to escape suppression, even while maintaining the TIP population. (The new restriction would decouple the relationship between η and the TIP population size.) This would not become evident for parameter range studied here until only very low numbers of TIPs were allowed (all plots were made restricting the number to be less than 200). However, for tight restrictions or very large η, the strategy does become viable.

References

[1] J.D. Bamdres, A.S. Shaw, and L. Ratner. Hiv-1 nef protein downregulation of cd4 surface expression: Relevance of the lck binding domain of cd4. *Virology*, 207:338–341, 1994.

[2] C. Fraser, T. D. Hollingsworth, R. Chapman, F. de Wolf, and W. P. Hanage. Variation in hiv-1 set-point viral load: epidemiological analysis and an evolutionary hypothesis. *Proc. Natl. Acad. Sci. U. S. A.*, 104(44):17441–6, 2007.

[3] Amy Hurford, Daniel Cownden, and Troy Day. Next-generation tools for evolutionary invasion analyses. *J R Soc Interface*, 7(45):561–571, Apr 2010.

[4] N. Jouvenet, S. M. Simon, and P. D. Bieniasz. Visualizing hiv-1 assembly. *J Mol Biol*, 410(4):501–11, 2011.

[5] V. T. Metzger, J. O. Lloyd-Smith, and L. S. Weinberger. Autonomous targeting of infectious superspreaders using engineered transmissible therapies. *PLoS Comput Biol*, 7(3):e1002015, 2011.

[6] H. Mohri, S. Bonhoeffer, S. Monard, A.S. Perelson, and D.D. Ho. Rapid turnover of t lymphocytes in siv-infected rhesus macaques. *Science*, 279:1223–1227, 1998.

[7] M. A. Nowak, A. L. Lloyd, G. M. Vasquez, T. A. Wiltrout, L. M. Wahl, N. Bischofberger, J. Williams, A. Kinter, A. S. Fauci, V. M. Hirsch, and J. D. Lifson. Viral dynamics of primary viremia and antiretroviral therapy in simian immunodeficiency virus infection. *Journal of virology*, 71(10):7518–25, 1997.

[8] A.S Perelson, D.E Kirschner, and R de Boer. Dynamics of hiv infection of cd4+ t cells. *Mathematical biosciences*, 114:81–125, 1993.

[9] I. M. Rouzine and L. S. Weinberger. Design requirements for interfering particles to maintain co-adaptive stability with hiv-1. *J. Virol.*, 87(4):2081–2093, 2013.

[10] I. M. Rouzine and LS. Weinberger. The quantitative theory of within-host viral evolution [review]. *J. Stat. Mech.: Theory and Experiment*, (1):P01009, 2013.

[11] Maria J Wawer, Ronald H Gray, Nelson K Sewankambo, David Serwadda, Xianbin Li, Oliver Laeyendecker, Noah Kiwanuka, Godfrey Kigozi, Mohammed Kiddugavu, Thomas Lutalo, Fred Nalugoda, Fred Wabwire-Mangen, Mary P Meehan, and Thomas C Quinn. Rates of hiv-1 transmission per coital act, by stage of hiv-1 infection, in rakai, uganda. *J Infect Dis*, 191(9):1403–1409, May 2005.
