# Supplementary figures and images for: Conflicting Selection Pressures Will Constrain Viral Escape from Interfering Particles: Principles for Designing Resistance-Proof Antivirals
Source: PLoS Comput Biol. 2016 May 6;12(5):e1004799. doi: 10.1371/journal.pcbi.1004799 (PMC4859541; doi:10.1371/journal.pcbi.1004799)

A

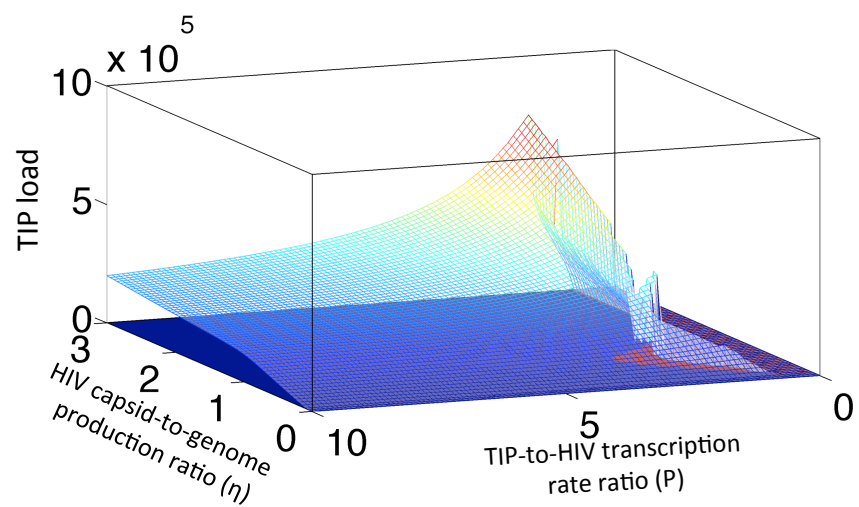

B

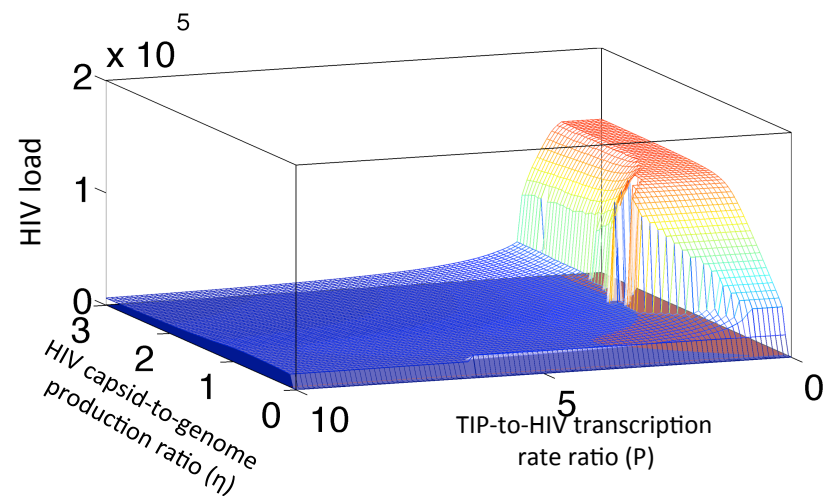

Supplement: S1 Fig — (A,B) Set-point levels of TIP and HIV within a host, measured in units of viremia (RNA copies/ml). The orange region in the X-Y plane denotes the region of within-host TIP instability. The average HIV viral load prior to TIP intervention is assumed to be 105. Model parameters are as listed in S1 Table. (PDF) [file pcbi.1004799.s002.pdf]

HIV+TIP- Prevalence

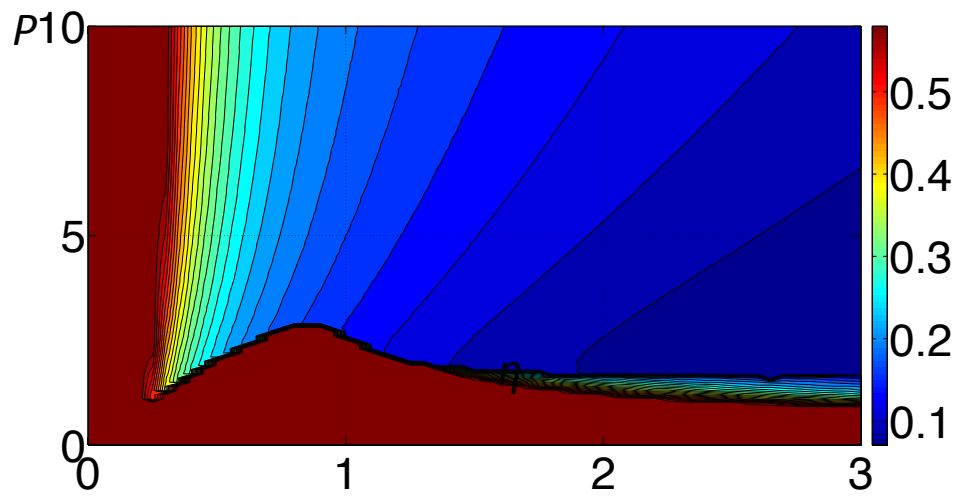

HIV Load (Within-host)

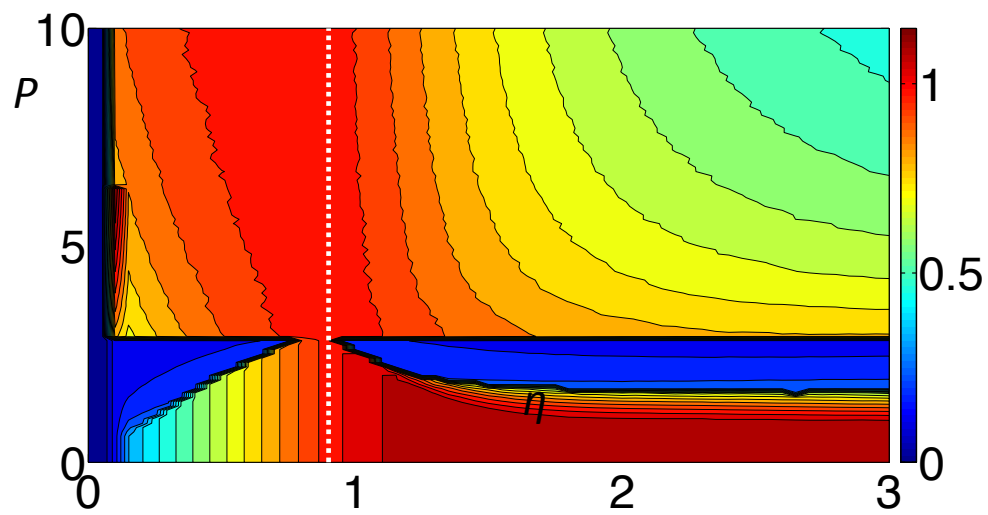

Supplement: S2 Fig — Heatmap analogs of Fig 2A expanded to include the region when P < 3. For each P, HIV load is normalized to its value at η = 0.9 (white dashed line). Here R0pop = 6.25; all other parameters are as shown in Tables 1 and 2. (PDF) [file pcbi.1004799.s003.pdf]

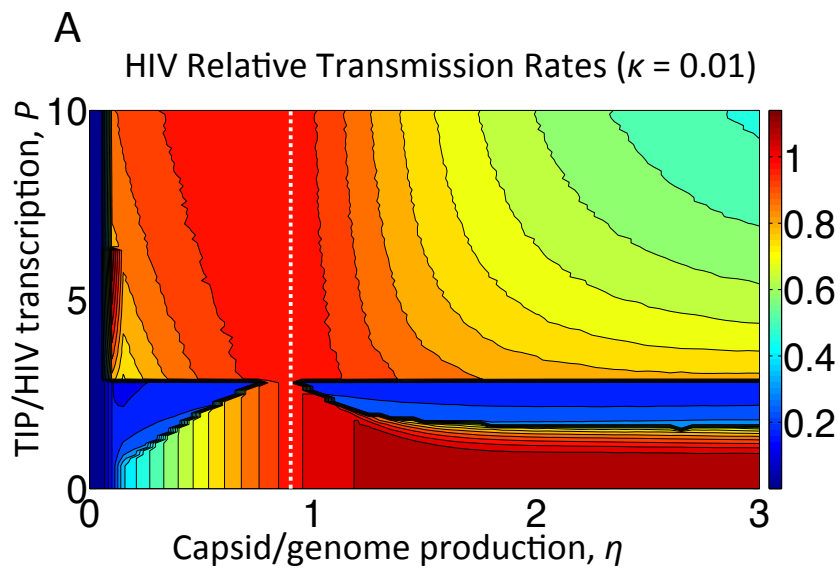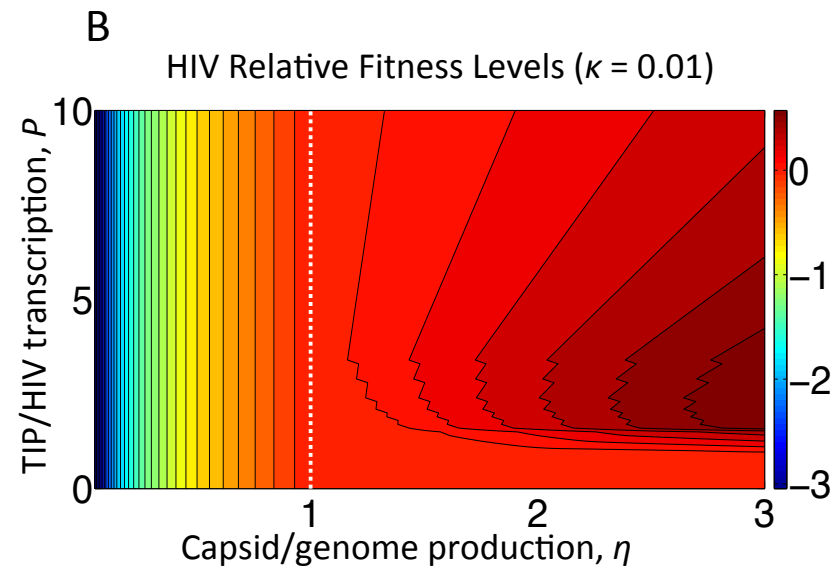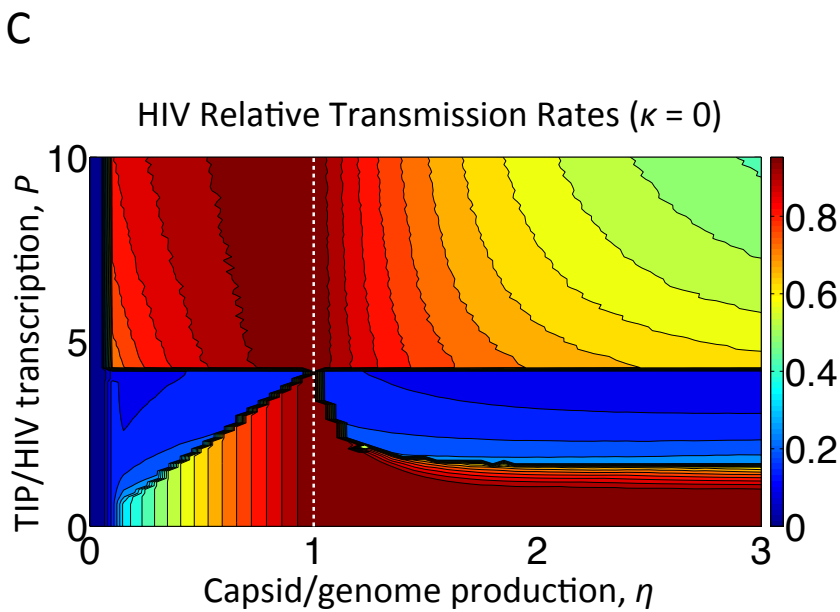

Supplement: S3 Fig — Heatmaps corresponding to Fig 2B in the main text. For each P in A-C, values are normalized to equal 1 along the vertical white dashed line. (A) Normalized HIV transmission rate as a function of η for each P, when the composite ‘waste’ parameter κ is set to equal 0.01 (see the Section A S1 Text or Table 2 for a definition of κ). (B) Normalized HIV fitness in individual hosts as a function of η for each P, when κ is again set to equal 0.01. While the transmission rate is highest at η close to 1, fitness increases monotonically with η (see also Fig 2B of the main text). This monotonic dependence on η occurs for any P, although the optimal transmission may vary slightly with P. (C) Relative transmission rate as a function of η for each P, with κ now set to equal 0. Note the reduced stability region as the peak shifts up and to the right. In these figures, R0pop = 6.25; all other parameters are as shown in Tables 1 and 2. (PDF) [file pcbi.1004799.s004.pdf]

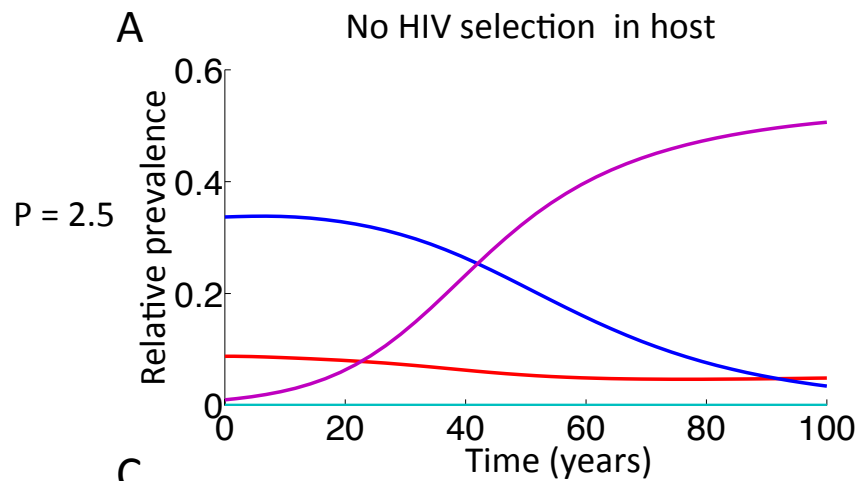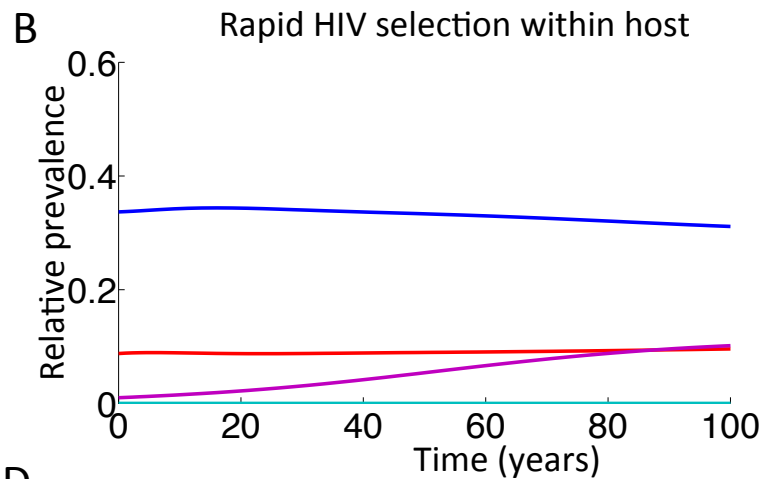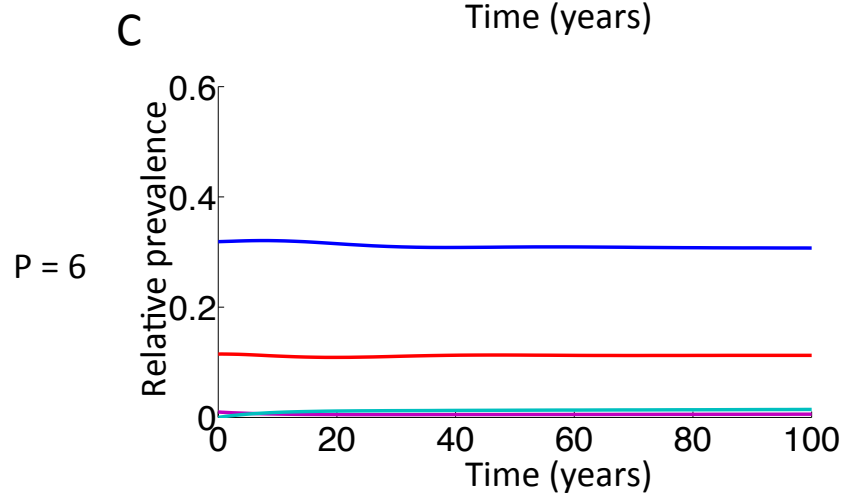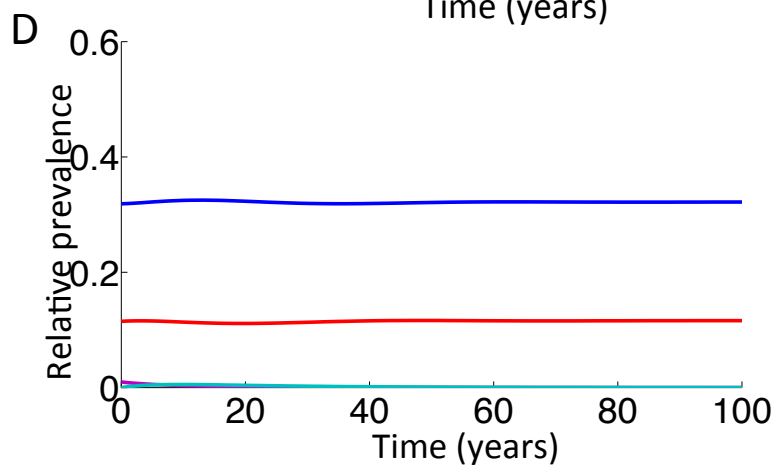

$I_D^{wt}$   
 $I_D^{mut}$   
 $\eta_{wt} = 2$   
 $\eta_{mut} = 1$

Supplement: S4 Fig — Prevalence of HIV and TIP sub-populations following the introduction of a TIP-resistant HIV mutant with ηmut = 1 into a population co-infected with TIP and a wild-type HIV strain with ηwt = 2. Four cases are shown: (A) P < Pc (i.e., P = 2.5) and the absence of HIV multi-strain co-infections in individual hosts, (B) P < Pc but the presence of HIV multi-strain co-infection, (C) P > Pc (i.e., P = 6) but the absence of HIV multi-strain co-infection, and (D) P > Pc and the presence of HIV multi-strain co-infection. When P < Pc (i.e., A, B), TIP-resistant mutants can make TIP interventions unstable; further, when co-infection is absent (i.e., A, C) TIP-resistant mutants can persist in the population. However, the combination of P > Pc and within-host co-infection (i.e., Panel D) results in the stability of TIPs against HIV mutation, and the extinction of TIP-resistant HIV mutants (due to within-host competitive exclusion by more-fit, higher η strains). See Tables 1 and 2 for a list of the fixed parameters and their values. (PDF) [file pcbi.1004799.s005.pdf]

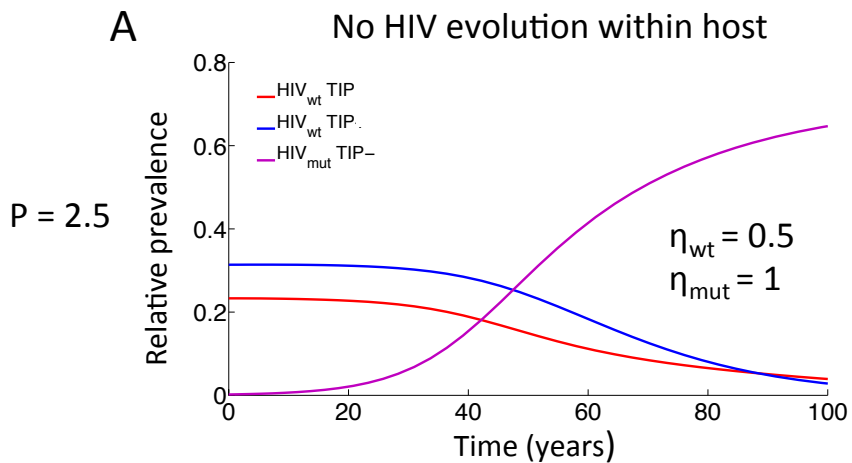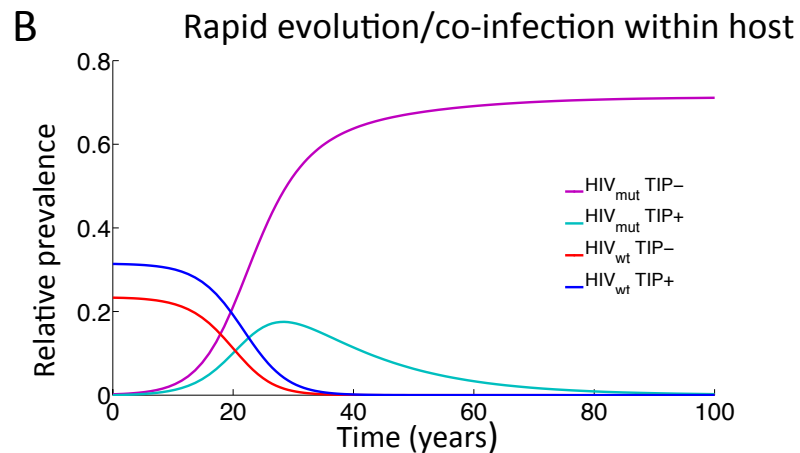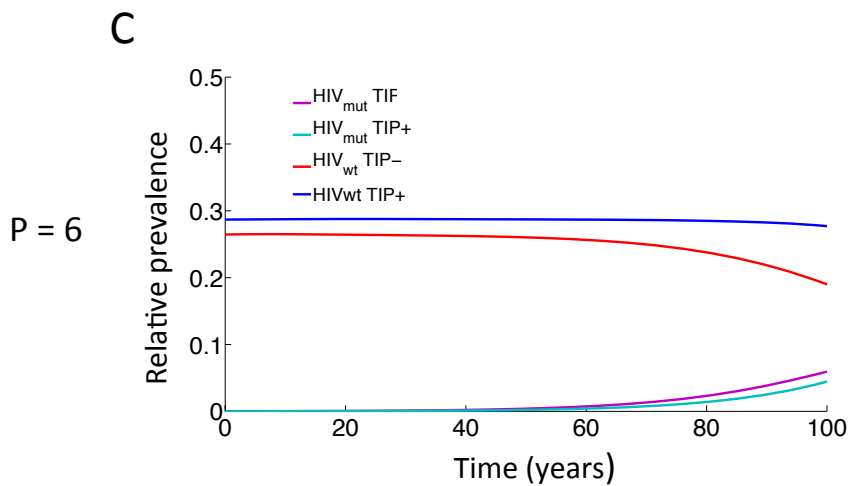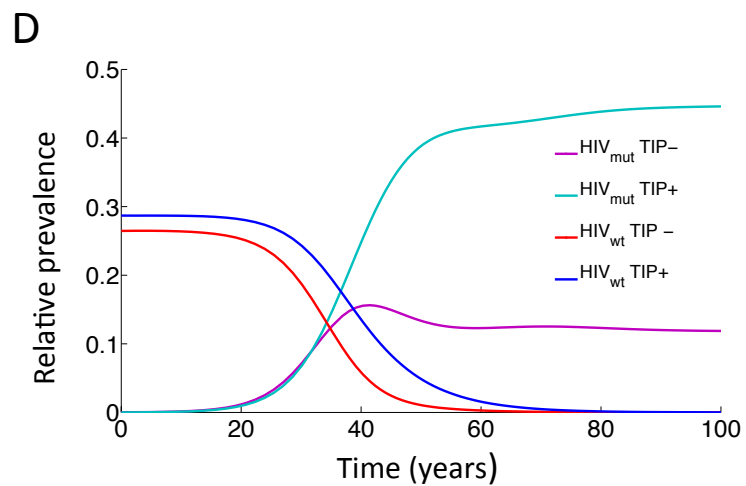

Supplement: S5 Fig — The same simulations as in the panels of S4 Fig, except that the wild type HIV strain now has a smaller capsid/genome ratio: ηwt = 0.5 instead of 1.5. The mutant strain still has ηmut = 1. Unlike in S4 Fig, there is no evolutionary conflict between the population scale and the host scale. Selection on both scales pushes HIV towards higher values of η. The presence of co-infection does not alter the direction of evolution, but only expedites the outcome. Still, P > Pc = 3 prevents TIP extinction when the HIV mutant invades, and in Panel D the final prevalence of singly-HIV infected individuals becomes less than before the mutant’s introduction. Parameters: R0pop = 6.25, ηwt = 0.5, ηmut = 1, P = 2.5 in (A,B) and P = 6 in (C,D). Tables 1 and 2 contain the remaining parameter values. (PDF) [file pcbi.1004799.s006.pdf]

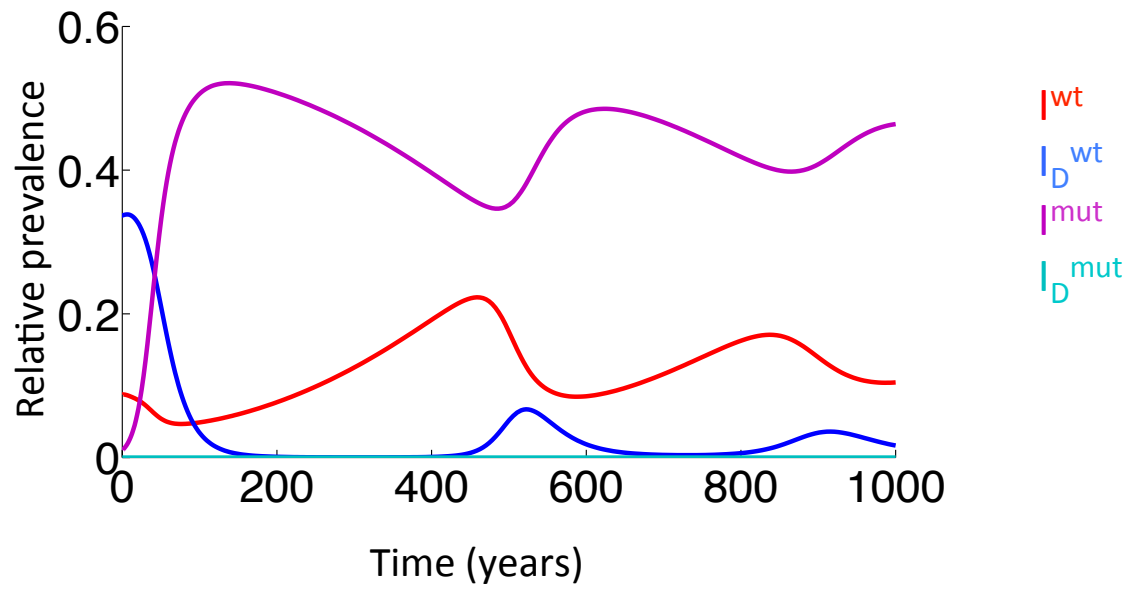

Supplement: S6 Fig — The relative fitness of the two HIV strains is dependent upon the prevalence of TIP infected hosts, which is in turn dependent on the relative frequencies of the two HIV strains. As a result, we see oscillations as the mutant takes over (see Discussion). Parameters: R0pop = 6.25, P = 2.5, ηwt = 2, ηmut = 1; all other parameters are contained in Tables 1 and 2. (PDF) [file pcbi.1004799.s007.pdf]

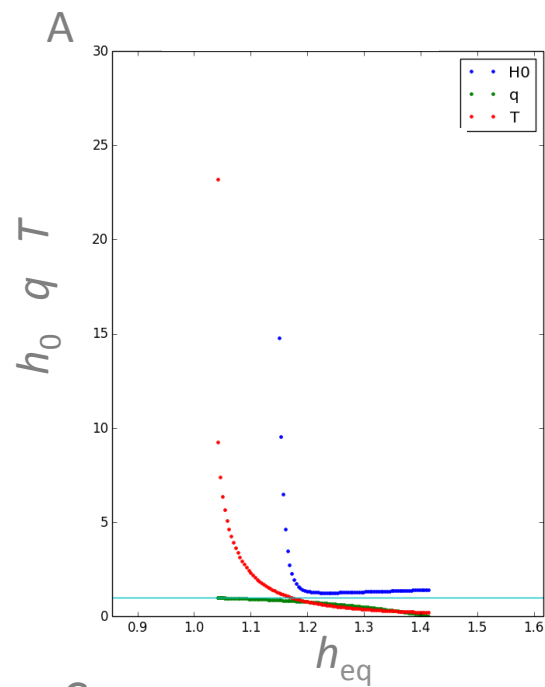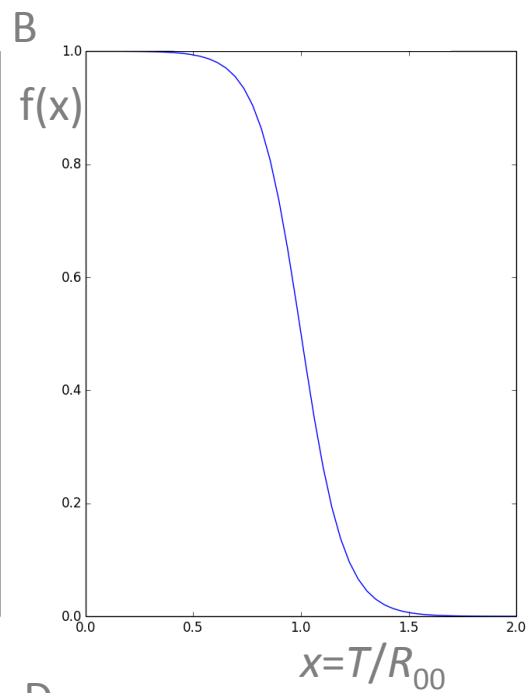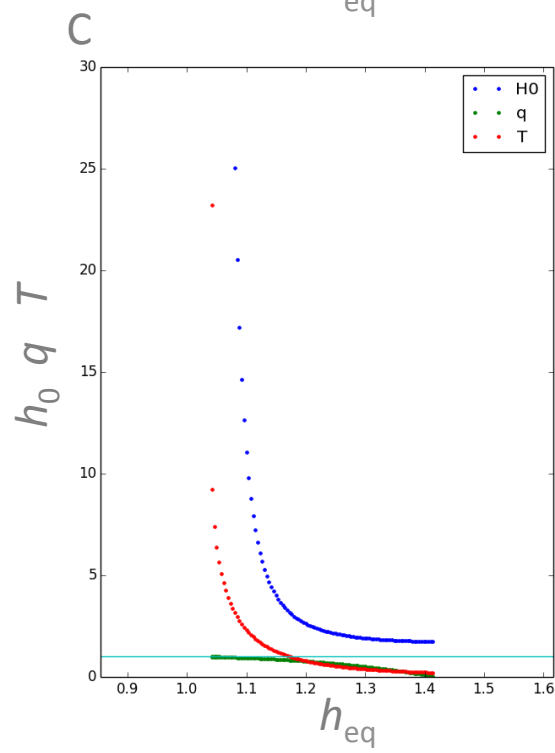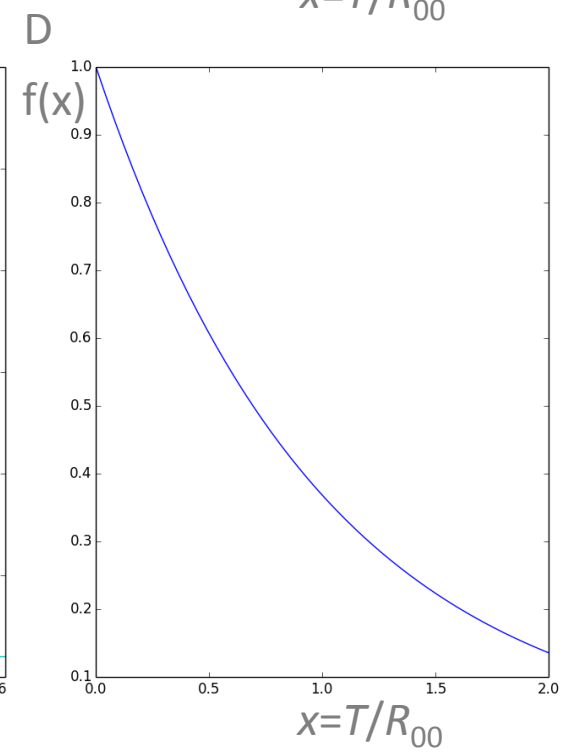

Supplement: S7 Fig — (A,C) The dependence of the maximum cell division rate (h0), relative target cell level (T), and mean TIP copy number per cell (q) on the equilibrium division rate heq (see Eqs. 51–58 in S1 Text). (B,D) Two forms of the homeostatic shutdown function. The dependence of heq on h0 is dominated by the asymptotic behavior of T as q approaches 1 (i.e., as the TIP copy number increases), and is robust to the shape of homeostatic shutdown function f(x). Parameters: η = 0.5, P = 12, R00 = 10; all other parameters are found in Table 2. (PDF) [file pcbi.1004799.s008.pdf]

A

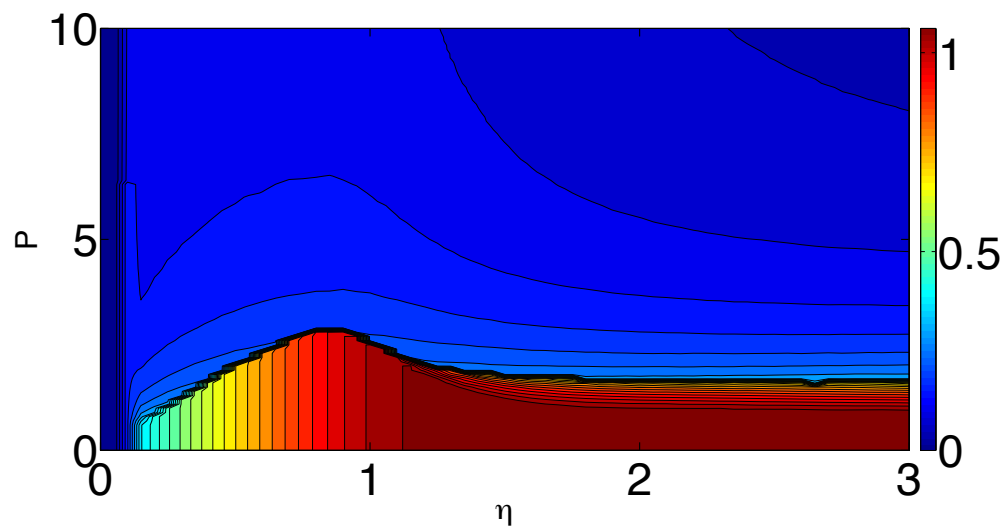

B

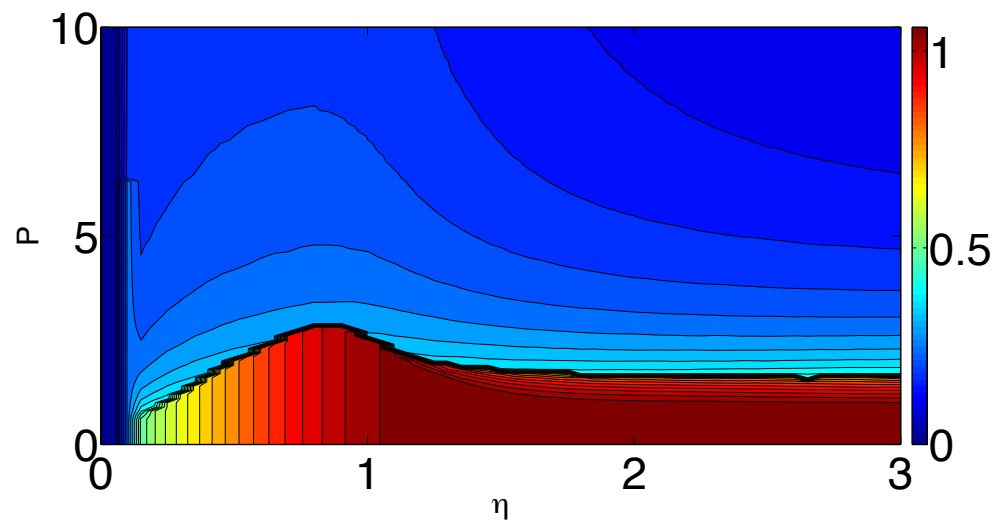

Supplement: S9 Fig — (A) Transmission potential assuming constant host death rates. (B) Transmission potential with changing death rates. (PDF) [file pcbi.1004799.s010.pdf]
