# Supplementary material for: Conflicting Selection Pressures Will Constrain Viral Escape from Interfering Particles: Principles for Designing Resistance-Proof Antivirals
Source: PLoS Comput Biol. 2016 May 6;12(5):e1004799. doi: 10.1371/journal.pcbi.1004799 (PMC4859541; doi:10.1371/journal.pcbi.1004799)
Supplement: S8 Fig — (A) TIP stability when HIV viral loads and transmission are assumed to suppressed by TIPs. (Left panel) HIV+TIP- prevalence, (middle panel) HIV+TIP+ prevalence, and (right panel) invasion analysis of HIV mutants performed as in Fig 3C. (B) TIP stability when HIV viral loads and transmission are assumed to be unsuppressed by TIPs. (Left panel) HIV+TIP- prevalence, (middle panel) HIV+TIP+ prevalence, and (right panel) invasion analysis of HIV mutants performed as in Fig 3C. Both the prevalence of HIV+, TIP- hosts and the evolutionary stability of TIPs remain virtually unchanged despite the absence of HIV suppression, because the additional transmission of HIV is matched by the increased spread of the TIP. (PDF) [file pcbi.1004799.s009.pdf]

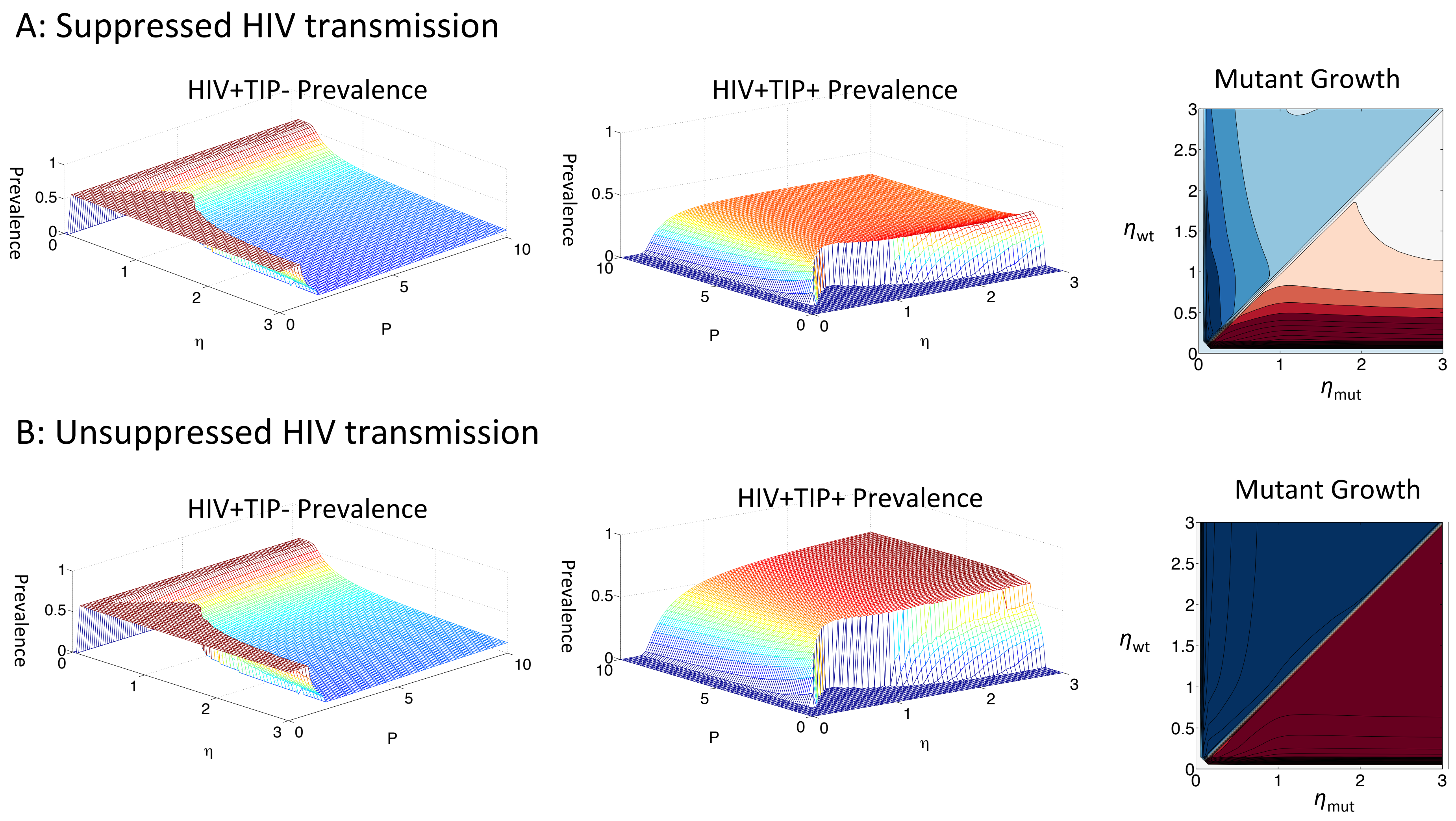

A: Suppressed HIV transmission
HIV+TIP- Prevalence
Prevalence
η
P
HIV+TIP+ Prevalence
Prevalence
P
η
Mutant Growth
η_{wt}
η_{mut}
B: Unsuppressed HIV transmission
HIV+TIP- Prevalence
Prevalence
η
P
HIV+TIP+ Prevalence
Prevalence
P
η
Mutant Growth
η_{wt}
η_{mut}
